# Endogenous CRISPR/Cas systems for genome engineering in the acetogens *Acetobacterium woodii* and *Clostridium autoethanogenum*

**DOI:** 10.3389/fbioe.2023.1213236

**Published:** 2023-06-23

**Authors:** Margaux Poulalier-Delavelle, Jonathan P. Baker, James Millard, Klaus Winzer, Nigel P. Minton

**Affiliations:** Clostridia Research Group, BBSRC/EPSRC Synthetic Biology Research Centre (SBRC), School of Life Sciences, Biodiscovery Institute, University of Nottingham, Nottingham, United Kingdom

**Keywords:** acetogen, *Acetobacterium woodii*, *Clostridium autoethanogenum*, endogenous CRISPR/Cas, protospacer adjacent motif (PAM), in-frame deletion

## Abstract

Acetogenic bacteria can play a major role in achieving Net Zero through their ability to convert CO_2_ into industrially relevant chemicals and fuels. Full exploitation of this potential will be reliant on effective metabolic engineering tools, such as those based on the *Streptococcus pyogenes* CRISPR/Cas9 system. However, attempts to introduce *cas9*-containing vectors into *Acetobacterium woodii* were unsuccessful, most likely as a consequence of Cas9 nuclease toxicity and the presence of a recognition site for an endogenous *A. woodii* restriction–modification (R-M) system in the *cas9* gene. As an alternative, this study aims to facilitate the exploitation of CRISPR/Cas endogenous systems as genome engineering tools. Accordingly, a Python script was developed to automate the prediction of protospacer adjacent motif (PAM) sequences and used to identify PAM candidates of the *A. woodii* Type I-B CRISPR/Cas system. The identified PAMs and the native leader sequence were characterized *in vivo* by interference assay and RT-qPCR, respectively. Expression of synthetic CRISPR arrays, consisting of the native leader sequence, direct repeats, and adequate spacer, along with an editing template for homologous recombination, successfully led to the creation of 300 bp and 354 bp in-frame deletions of *pyrE* and *pheA*, respectively. To further validate the method, a 3.2 kb deletion of *hsdR1* was also generated, as well as the knock-in of the fluorescence-activating and absorption-shifting tag (FAST) reporter gene at the *pheA* locus. Homology arm length, cell density, and the amount of DNA used for transformation were found to significantly impact editing efficiencies. The devised workflow was subsequently applied to the Type I-B CRISPR/Cas system of *Clostridium autoethanogenum*, enabling the generation of a 561 bp in-frame deletion of *pyrE* with 100% editing efficiency. This is the first report of genome engineering of both *A. woodii* and *C. autoethanogenum* using their endogenous CRISPR/Cas systems.

## 1 Introduction

In view of the current climate crisis, Net Zero defines the global commitment to cut greenhouse gas (GHG) emissions to as close to zero as possible by 2050. It is recognized that GHG emissions associated with the continued exploitation of fossil resources are the main driver of climate change. Therefore, innovative solutions for production of fuels and chemicals that participate in atmospheric CO_2_ reduction are required.

The Wood–Ljungdahl Pathway (WLP) allows acetogenic bacteria to grow on CO_2_ as their sole carbon source. This ancient pathway supports the conversion of two molecules of CO_2_ to one molecule of acetyl-CoA, which may then be converted to different C_2_-products (acetate or ethanol) or elongated to C_4_ (butyrate) or C_5_-products (caproate), depending on the species (Schuchmann and Muller, 2016). Acetogens, therefore, represent a particularly attractive option for achieving Net Zero. *Clostridium autoethanogenum*, for instance, produces ethanol in addition to acetate and now forms the basis of a commercial process for its production from steel mill off-gas (Liew et al., 2016). However, for yield improvements and extension of the range of chemicals that can be produced by acetogens, effective genome editing tools are required.

In recent years, genome editing systems based on the Type II CRISPR/Cas9 system of *Streptococcus pyogenes* (SpCas9) (Nishimasu et al., 2014) have played a pivotal role in genetic engineering advances. They have simplified the rapid generation of markerless knock-out (KO), knock-in (KI), and point mutations in multiple bacterial species (Wang et al., 2015; Li et al., 2016; McAllister and Sorg, 2019), including acetogens like *Clostridium ljungdahlii* and *C. autoethanogenum* (Huang et al., 2016; Seys et al., 2020). Despite their benefits, these systems have many drawbacks, most notably poor transfer rates due to the large size of the encoding *cas9* gene and its toxicity when expressed in cells.

Among acetogens, *A. woodii* is a homoacetogen, in that it primarily produces acetate under autotrophic and heterotrophic conditions (Schuchmann and Muller, 2016). Attempts to use the SpCas9-based RiboCas system (Canadas et al., 2019) in *A. woodii* yielded low transformation efficiencies, despite the *cas9* gene being under tight regulatory control. Preliminary experiments indicated that nuclease toxicity and a Type I restriction-modification system, a N^6^-adenine DNA methyltransferase (encoded by AWO_c08800, AWO_c08810, and AWO_c08870) with target sequence 5′-TAAGN_5_TCC-3′, were hindering strain transformation (data not shown). Accordingly, an alternative method, not reliant on the *S. pyogenes* Cas9, was required for gene editing in *A. woodii*. CRISPR systems are present in approximately 45% of sequenced bacterial genomes (Grissa et al., 2007a; Rousseau et al., 2009), which has led to the creation and improvement of databases of predicted CRISPR loci and tools to allow prediction of CRISPR loci in newly sequenced genomes (Grissa et al., 2007a; Grissa et al., 2007b; Rousseau et al., 2009; Couvin et al., 2018). As a result, endogenous CRISPR/Cas systems are becoming better characterized and represent an attractive basis for the development of gene editing systems in many bacteria. In *Clostridium* spp*.*, 74% of strains have an endogenous CRISPR system (Pyne et al., 2016), and endogenous Type I-B systems have successfully been used to genetically modify *Clostridium pasteurianum* (Pyne et al., 2016), *Clostridium butyricum* (Zhou et al., 2021)*, Clostridioides difficile* (Maikova et al., 2019), and *Clostridium thermocellum* (Walker et al., 2020).

In Type I systems, the CRISPR locus is composed of a Cas cluster and one or more CRISPR arrays. The Cas cluster contains all the Cas subunits, including the subunits of the Cascade effector complex necessary for interference; the array(s) contain multiple spacers separated by identical direct repeats (DRs) (Nishimasu and Nureki, 2017). A leader sequence controls the expression of CRISPR arrays and is usually located between the cluster and the array (Zhang et al., 2018; Moon et al., 2019). The spacers are derived from previous invader sequences and allow sequence-specific targeting of new invaders. The array is transcribed into pre-crRNA and further processed by the Cas machinery to form mature crRNA. Foreign DNA can be targeted when it contains a sequence matching one of the spacers and an appropriate protospacer adjacent motif (PAM) to distinguish it from endogenous DNA (Nishimasu and Nureki, 2017). In the presence of a protospacer and a PAM, the Cascade–crRNA complex binds to the target DNA, and the Cas3 nuclease is recruited (Mulepati and Bailey, 2013). Cas3 nicks both strands of DNA upstream of the protospacer (Mulepati and Bailey, 2013). It was found that the interference process is directed by a seed sequence adjacent to the PAM rather than the full spacer (Semenova et al., 2011; Wiedenheft et al., 2011), possibly enabling faster screening of DNA entering the cells. It was also shown that in the genome, new spacers are inserted at the beginning of the CRISPR array (Alkhnbashi et al., 2016) by a Cas1–Cas2 complex, making the new spacers likely to have a relatively higher level of expression.

This study describes the steps undertaken to facilitate the exploitation of endogenous CRISPR/Cas systems as genome engineering tools in two acetogens, *A. woodii* and *C. autoethanogenum*. A Python script was developed for PAM prediction and validated against previously published functional PAMs. *A. woodii* Type I-B endogenous CRISPR/Cas system was analyzed, an interference assay confirmed potential PAMs, and RT-qPCR confirmed the functionality of the leader sequence. For genome engineering, the requisite knock-out vectors were built with two components, a synthetic array and an editing template. The synthetic array mimics native arrays; it contains one spacer flanked by direct repeats and is under the control of the native leader sequence. The editing template consists of two homology arms (HAs) to allow homologous recombination. Knock-outs and a reporter gene knock-in were achieved in *A. woodii* while validating different parameters to improve editing efficiency. Subsequently, the workflow was successfully applied to *C. autoethanogenum*, demonstrating the transferability of the techniques presented.

## 2 Materials and methods

### 2.1 Python script development

The Python script described in Supplementary Figures S1, S2 was created in the PyCharm IDE (Integrated Development Environment) with Biopython modules (the Python script will be made available upon request). An object called Spacer was created to easily compile all the information collected on each hit for each spacer (See Supplementary Table S1 for a full list of attributes collected) and to facilitate submission, retrieval, and filtering of information from databases. INPUT—the user needs to enter an identification email and the GenBank accession number of the studied organism, as well as a set of information about the CRISPR system which can be retrieved from the CRISPRFinder database (Grissa et al., 2007a; Grissa et al., 2007b; Couvin et al., 2018). ARRAY—the array direction is set either with the CRISPRFinder predictions or an internal function submitting the flanks to BPROM (Solovyev and Salamov, 2011). If no orientation is found, the user is prompted to enter an orientation. BLAST—the protospacer list is submitted to BLASTn with a filtering function: global mismatch lower than 20% and no more than one mismatch in the first seven nucleotides. PHASTER—information on the hits is retrieved to update the phage attribute, whether directly from the position of the hits or after submission to PHASTER (Zhou et al., 2011; Arndt et al., 2019). PAM—if at least two of the hits are in phage regions, a consensus sequence and WebLogo graphical representations are then created to represent the potential PAMs (Crooks et al., 2004). OUTPUT—confirmation of intermediate steps is displayed in the console, and the final output of the script is an Excel summary document.

### 2.2 Chemicals

All the chemicals were purchased from Sigma-Aldrich, unless otherwise stated.

### 2.3 Bacterial strains and plasmids

The following strains were used: *Escherichia coli* TOP10 (Invitrogen), wild-type (WT) *A. woodii* strain DSM1030 (DSMZ), and WT *C. autoethanogenum* strain DSM10061 (DSMZ). Strains are listed in Supplementary Table, S2.

### 2.4 Growth media and conditions

*E. coli* was grown aerobically at 37°C in LB medium supplemented with the appropriate antibiotic: chloramphenicol 25 μg/mL in plates or 12.5 μg/mL in liquid cultures and spectinomycin 250 μg/mL. *A. woodii* was grown anaerobically at 30°C in either liquid *A. woodii* medium (AWM) or modified ATCC medium 1019 plates, supplemented when required with thiamphenicol 15 μg/mL. For phenotypic work, ATCC medium lacking yeast extract was used supplemented, when required, with uracil or phenylalanine at 20 μg/mL. The trace element solution SL9, the selenite–tungstate solution, and the vitamin solution were prepared separately and stored at 4°C. *C. autoethanogenum* was grown anaerobically at 37°C in YTF media supplemented with the appropriate antibiotic: 15 μg/mL thiamphenicol in plates or 7.5 μg/mL in liquid cultures and 250 μg/mL D-cycloserine in plates. For phenotypic work, PETC MES agar plates were used, supplemented when required with 20 μg/mL uracil. The list of ingredients for the media and the different solutions is given in Supplementary Tables S3–S5.

### 2.5 DNA manipulations

All the primers used for the constructs are listed in Supplementary Table S6. Some of the plasmid backbones were taken from the culture collection of the laboratory. All the plasmids built were built according to the pMTL80000 shuttle plasmid standards (Heap et al., 2009). All plasmids used are listed in Supplementary Table S7. The Monarch Plasmid Miniprep Kit, Monarch DNA Gel Extraction Kit, Q5 High-Fidelity Master Mix, restriction enzymes, and T4 DNA Ligase were purchased from New England Biolabs and used according to the manufacturer’s protocols. The plasmids required for this study and the colony PCRs to verify their assembly or their presence are described hereafter. Colony PCRs to verify the corresponding genome locus in *A. woodii* or *C. autoethanogenum* are also described.

#### 2.5.1 Interference assay plasmids

The following primer pairs were annealed with Q5: FW_proto8/RV_proto8, FW_proto8-5′/RV_proto8, FW_proto20/RV_proto20, FW_proto20-5′/RV_proto20, FW_proto22/RV_proto22, and FW_proto22-5′/RV_proto22 to yield the three protospacers with and without their PAM candidate. They were then inserted by HiFi in a SacII/SalI-digested pMTL82151 backbone to create pMTL-MPD1 to 6. Colony PCRs and Sanger sequencing were performed with primers ColE1+tra-F2 and pBP1-R1.

#### 2.5.2 Endogenous leader characterization

The MCS from pMTL82151 was inserted after the leader sequence in pMTL-MPD21 by digestion with SacI/NheI and ligation with T4 DNA Ligase, creating pMTL-MPD15 (the full sequence of the vector pMTL-MPD15 is available at www.plasmidvectors.com (RRID: SCR_023475), where it may be sourced). The FAST gene and the *catP* gene were inserted in the MCS after the leader sequence for characterization of the leader sequence by RT-qPCR and to function as a negative control, respectively. The *catP* and FAST genes were amplified with primer pairs FW_catP_SacI/RV_catP_NheI and FW_FAST_SacI/RV_FAST_NheI, respectively. The pMTL-MPD15 backbone and both genes were digested by SacI and NheI. The *catP* and FAST genes were ligated in the backbone with T4 DNA ligase (NEB), yielding pMTL-MPD30 and pMTL-MPD29, respectively. All plasmids were confirmed by Sanger sequencing with primers ColE1+tra-F2 and pCD6-R1.

#### 2.5.3 *pyrE* KO in *A. woodii*

The editing template consisted of two homology arms of 471 bp (LHA) and 491 bp (RHA). The spacer was designed to be 36 bp and at a distance of 29 bp from the RHA. Two Gram-positive replicons were selected, pBP1 and pCD6.

The *cas9* gene, together with its P_*fdxE*_ promoter, was removed from pMTL8215_pRECas1_MCS (Canadas et al., 2019) by XbaI/NotI digestion, blunting, and re-ligation, with T4 DNA polymerase and T4 DNA Ligase, respectively, yielding the vector pMTL8215_P1339_MCS. The editing cassette was assembled in this plasmid. HAs were amplified with primer pairs FW_LHA_pyrE/RV_LHA_pyrE+BM4 and FW_BM4+1+RHA_pyrE/RV_RHA_pyrE from *A. woodii* genomic DNA, assembled by SOEing PCR (Splicing by Overlap-Extension PCR), and ligated into the vector after AscI/AatII digestion, creating pMTL-MPD7.

*A. woodii* leader sequence was amplified with primers FW_leader/RV_leader; the synthetic array was created by annealing primers FW_pyrE_endo2 and RV_pyrE_endo2. Both the leader sequence and synthetic array were assembled by HiFi into pMTL-MPD7 digested by SacII/AatII, yielding pMTL-MPD8.

The editing cassette from pMTL-MPD8, amplified with primers FW_leader and RV_RHA_pyrE+MCS, pMTL82151, and pMTL84151, were digested by AatII/NheI, SacII/AatII, or SacII/NheI and ligated together to obtain constructs with HAs alone, expressed spacer alone, or the whole editing cassette, respectively, with either Gram-positive replicon, yielding plasmids pMTL-MPD9 to 14.

Colony PCRs were performed with primers ColE1+tra-F2/pBP1-R1 or ColE1+tra-F2/pCD6-R1 and plasmids confirmed by Sanger sequencing. The same primers were used to check for plasmid presence in *A. woodii,* and the *pyrE* locus was analyzed with primers AW_CRISPR_pyrE_armF and AW_pyrEcomp_RHAR.

#### 2.5.4 *pheA* KO in *A. woodii* with 500 bp, 1.0 kb, and 1.5 kb HA

The spacer was designed to be 35 bp and at a distance of 26 bp from the RHA. Homology arms of 0.5, 1.0, and 1.5 kb were designed. The resulting editing cassettes were inserted into a backbone with the pCD6 Gram-positive replicon.

The spacer sequence was created by annealing primers FW_pheA_spacer and RV_pheA_spacer by PCR and digestion by SacI and AatII; the leader sequence was created by amplification with FW_leader/RV_leader and SacII and SacI digestion; both fragments were assembled in pMTL84151 digested by SacII/AatII. Homology arms of 0.5 kb, 1.0 kb, and 1.5 kb were designed. Left homology arms were amplified with primers FW_L0.5/L1.0/L1.5 and RV_LHA_pheA; right homology arms were amplified with primers FW_RHA_pheA and RV_R0.5/R1.0/R1.5. The LHA and RHA were assembled by SOEing PCR with primer pairs FW_L0.5/RV_R0.5, FW_L1.0/RV_R1.0, and FW_L1.5/RV_R1.5. Assembled HAs were ligated in both pMTL84151 and pMTL84151 containing the assembled leader and spacer after AatII/NheI digestion, yielding three plasmids containing the HAs alone (pMTL-MPD16 to 18) and three editing plasmids containing the expressed spacer and the HAs (pMTL-MPD19 to 21).

Colony PCRs were performed with primers ColE1+tra-F2/pCD6-R1 and plasmids confirmed by Sanger sequencing. *A. woodii* colonies were analyzed by colony PCR both for plasmid presence with primers ColE1+tra-F2 and pCD6-R1 and for the *pheA* locus with primers FW_pheAHA1.5_out and RV_pheAHA1.5_out.

#### 2.5.5 *hsdR1* KO in *A. woodii*

Homology arms of 1,455 bp (LHA) and 1,489 bp (RHA) and a 36 bp spacer starting 40 bp from the LHA were designed.

Primers FW_hsdR1_spacer and RV_hsdR1_spacer were annealed by PCR and inserted in the pMTL84151 vector with the assembled leader and spacer used for *pheA* KO by SacI and AatII digestion and ligation with T4 DNA Ligase, resulting in the vector pMTL8415_leader_hsdR1_spacer. The left and right homology arms were amplified with the primers FW_LHA_hsdR1/RV_LHA_hsdR1 and FW_RHA_hsdR1/RV_RHA_hsdR1, respectively, and assembled by SOEing PCR with the FW_LHA_hsdR1/RV_RHA_hsdR1 primer pair. The vector containing the spacer and the assembled HAs were digested by AatII and NheI and ligated together with T4 DNA Ligase, resulting in the final editing vector for *hsdR1* KO, pMTL-MPD22.

Colony PCRs were performed with primers ColE1+tra-F2/pCD6-R1 and plasmids confirmed by Sanger sequencing. Cell densities and the amount of DNA were investigated as potential factors to increase editing efficiency as follows: 100 µL of cells and 1 µg of DNA (T1); 100 µL of high-density cells and 1 µg of DNA (T2); 200 µL of high-density cells and 4 µg of DNA (T3). *A. woodii* colonies were analyzed by PCR for plasmid presence with primers ColE1+tra-F2/pCD6-R1 and the *hsdR1* locus with primers FW_hsdR1_screen_1.5/RV_hsdR1_screen_1.5 and FW_hsdR1_del_screen/RV_hsdR1_del_screen.

#### 2.5.6 FAST gene KI at the *pheA* locus in *A. woodii*

The spacer is 36 bp, and homology arms are 1,496 bp and 1,465 bp and were designed for insertion of the cargo in place of the PAM. The cargo consists of the FAST reporter gene flanked by the thiolase (*thl*) promoter (P_*thl*_) and *pyrE-hydA* (orotate phosphoribosyltransferase and hydrogenase I) terminator from *C. acetobutylicum* and was inserted between the homology arms.

Primers FW_AWO_pheA_CCG_KI_spacer and RV_AWO_pheA_CCG_KI_spacer were annealed by PCR and inserted in the pMTL84151 vector with the assembled leader and spacer used for *pheA* KO by SacI and AatII digestion and ligation with T4 DNA Ligase. The left and right homology arms were amplified with primers FW_AWO_LHA_pheA_cargo/RV_AWO_LHA_pheA_CCG_cargo_MCS and FW_AWO_RHA_pheA_CCG_cargo_MCS/RV_AWO_RHA_pheA_cargo, respectively, and assembled by SOEing-PCR with the FW_AWO_LHA_pheA_cargo/RV_AWO_RHA_pheA_cargo primer pair. The assembled HAs were inserted in the vector containing the spacer by AatII and NheI digestion and ligation by T4 DNA Ligase, resulting in a KI vector (pMTL-MPD23) containing an MCS with BamHI, NotI, NdeI, and XbaI restrictions sites between the two homology arms.

A P_*thl*__FAST_term cassette was amplified with the primer pair FW_BamHI_FAST_cassette/RV_FAST_NotI_AscI from plasmid pMTL8415X_P_*thl*__FAST with the FAST gene flanked by the *thl* promoter and *pyrE-hydA* terminator. The cassette was then inserted into the MCS of the pMTL-MPD23 vector by XbaI and NotI digestion and ligation with T4 DNA Ligase, yielding pMTL-MPD24.

Plasmids were confirmed by Sanger sequencing with ColE1+tra-F2/pCD6-R1/FW_pheA_cargo_seq/RV_pheA_cargo_seq.

WT *A. woodii* was transformed with the final editing vector, pMTL-MPD24, following the protocol named T3 in the previous section, with 200 µL of high-density cells and 4 µg of DNA. *A. woodii* colonies were analyzed by colony PCR for plasmid presence with primers ColE1+tra-F2/pCD6-R1 and the *pheA* locus with primers FW_pheA_KI_seq and RV_pheA_KI_seq.

#### 2.5.7 *pyrE* KO in *C. autoethanogenum*

Homology arms of 868 bp and 797 bp were used, and the synthetic array was designed to mimic *C. autoethanogenum* native array 4, with the native leader sequence controlling the expression of a 35 bp spacer flanked by direct repeats. The editing cassette was inserted into a pMTL83151 backbone.

pMTL83151 and CRISPR array leader amplified with FW_leader4_CLAU/RV_leader4_CLAU were digested by SacII/SacI and ligated together to yield pMTL-MPD25 (the full sequence of the vector pMTL-MPD25 is available at www.plasmidvectors.com (RRID: SCR_023475), where it may be sourced). Primers FW_CLAUpyrE_spacer1 and RV_CLAUpyrE_spacer1 were annealed by PCR and inserted into pMTL-MPD25 by SacI and BamHI digestion and ligation with T4 DNA Ligase, yielding pMTL-MPD27. This plasmid and the homology arms amplified with FW_CLAUpyrE_HA/RV_CLAUpyrE_HA from plasmid vFS67 (provided by Dr. Francois Seys) were digested by BamHI/NheI and ligated together to yield the final editing vector, pMTL-MPD28. An intermediate vector (pMTL-MPD26) containing the HAs alone was obtained by digesting pMTL83151 and the HAs by BamHI/NheI and ligation.

Colony PCRs were performed with primers ColE1+tra-F2 and pCB102-R1 and plasmids confirmed by Sanger sequencing. *C. autoethanogenum* colonies were analyzed by PCR for plasmid presence with ColE1+tra-F2 and pCB102-R1 and the *pyrE* locus analyzed with FW_pyrECLAU_screening and RV_pyrECLAU_screening.

### 2.6 Strain engineering

#### 2.6.1 *Escherichia coli*

##### 2.6.1.1 Chemically competent cells

An overnight culture of *E. coli* TOP10 was used to inoculate 100 mL of LB medium. Once the OD_600nm_ reached 0.4–0.5, the culture was incubated at 4°C for 30 min. The cells were then collected by centrifugation at 5,000 rpm for 5 min at 4°C*.* The cells were washed and incubated for 15 min with ice-cold 0.1 M MgCl_2_. A second wash and incubation was performed with ice-cold 0.1 M CaCl_2_. Finally, the cells were resuspended in 1 mL of ice-cold 0.1 M CaCl_2_ with 20% glycerol. The cells were then aliquoted for storage and kept at −80°C until further use.

##### 2.6.1.2 Transformation and colony PCR

A volume of 50 µL of chemically competent *E. coli* TOP10 cells was incubated with DNA for 30 min on ice, then 30 s at 42°C, 5 min on ice, and a final 1 h recovery in SOC medium before plating on selective LB agar plates. Colonies were checked by colony PCRs to verify the presence of the plasmids using Taq with standard buffer (New England Biolabs). The plasmids were then extracted using the Monarch Plasmid Miniprep Kit (New England Biolabs) and confirmed by Sanger sequencing.

#### 2.6.2 *Acetobacterium woodii*

##### 2.6.2.1 Competent cells: standard and high density

A 48 h liquid culture of the appropriate strain of *A. woodii* was used to inoculate a 300 mL culture to reach an OD_600nm_ of between 0.3 and 0.4 overnight. The cells were collected and then washed twice with filter-sterilized SMP5.8 buffer (1 mM sodium phosphate pH 5.8, 1 mM MgCl_2_, and 270 mM sucrose). In the final step, they were resuspended in 2 mL of SMP5.8 buffer for standard competent cells or in 666 µL for high-density competent cells, and 100% DMSO was added to reach 10% concentration. The cells were aliquoted for storage and kept at −80°C until further use.

##### 2.6.2.2 Transformation and colony PCR

*A. woodii* was transformed by electroporation using either 100 µL of competent cells and 1 µg of plasmid in 20 µL of water or 200 µL of high-density competent cells and 4 µg of plasmid in 20 µL of water. Electroporation was performed using a Gene Pulser Xcell™ electroporator (Bio-Rad). After 5 h (unless stated otherwise) of recovery in AWM, transformations were plated on modified AWM containing the appropriate selection or supplementation. After 10 days, the transformation efficiencies were recorded. The colonies were then cultured in liquid AWM, and after 48 h, colony PCRs were performed for analyzing plasmid presence and genome locus, when appropriate, using Taq DNA polymerase (NEB). The PCRs were subsequently confirmed by Sanger sequencing.

##### 2.6.2.3 Plasmid loss

After mutant strain confirmation, a series of inoculations into liquid broth and agar plates were performed for plasmid loss. Single colonies were streaked on plates with and without selection to check for plasmid loss.

#### 2.6.3 *Clostridium autoethanogenum*

##### 2.6.3.1 Conjugation and colony PCR

YTF was inoculated to an OD_600nm_ of 0.02 from a late exponential (48–72 h) preculture of the *C. autoethanogenum* recipient strain and incubated overnight. An *E. coli* NEB sExpress conjugation donor (Woods et al., 2019) harboring the shuttle vector was inoculated and incubated overnight in LB supplemented with spectinomycin and additional antibiotics for the shuttle vector.

The next day, LB broth containing the appropriate antibiotic selection was inoculated with the *E. coli* donor strain and incubated until OD_600nm_ 0.2–0.4 was reached. A 1 mL aliquot of the *E. coli* donor strain culture was collected at 3,000 g for 3 min, washed in 500 µL of PBS through gentle flicking of the tube, and centrifuged again at 3,000 g for 3 min. The pellet was then transferred into an anaerobic cabinet and resuspended in 200 µL of the overnight culture of *C. autoethanogenum*. The mixture was spotted onto non-selective YTF agar plates and incubated overnight (16–24 h on a mating plate).

The next day, the growth on the non-selective mating plate was collected using 500 µL of anaerobic PBS and a wedge-shaped spreader. The slurry was then spread onto agar plates with appropriate selection for the shuttle vector and D-cycloserine as an *E. coli* counter-selection. The plates were incubated under anaerobic conditions for 3–4 days. Once the colonies were visible, they were inoculated onto fresh agar plates with selection, and single colonies were inoculated in liquid cultures with appropriate antibiotic. Glycerol stocks and colony PCRs were performed after 48 h using Taq DNA polymerase (NEB). The PCRs were subsequently confirmed by Sanger sequencing.

##### 2.6.3.2 Plasmid loss

After mutant strain confirmation, a series of inoculations into liquid broth and agar plates were performed for plasmid loss. Colonies were inoculated onto non-selective plates, either directly from the liquid cultures with selection used for colony PCRs, or from a subculture of it without selection. From those non-selective plates, single colonies were streaked onto non-selective plates. Single colonies were streaked on plates with and without selection to check for plasmid loss.

### 2.7 Flow cytometry

*A. woodii* cells were grown until the early- to mid-exponential phase and then pelleted at 7,000 g for 5 min. The cells were washed twice in PBS before being resuspended in PBS. A volume of 100 μL of 25 µM ^TF^Amber (The Twinkle Factory) or PBS was added to 400 µL of resuspended cell samples immediately before fluorescence analysis. Cell fluorescence was analyzed using an Astrios EQ cell sorter (Beckman Coulter) with a 488 nm laser and a 576/21 nm filter. Data were analyzed using the Kaluza 2.1 analysis software (Beckmann Coulter). The population was gated to eliminate outlier cells and obtain consistent results for each sample, a first gate to select cells and a second gate to select single cells when represented on a side scatter/forward scatter plot. The percentage of cells gated was represented as a function of the FAST Amber fluorescence intensity. The number of events for each graph is as follows: WT without/with ligand: 549,749/441,371; plasmid without/with ligand: 2,568,561/6,791,597; KI without/with ligand: 1,285,872/5,630,864.

### 2.8 Endogenous leader characterization

WT *A. woodii* was separately transformed with the plasmids described in Section 2.5.2, colonies were grown in liquid cultures, and samples were taken at different stages of cell growth. RNA was extracted, and RT-qPCR was performed as described in the following sections.

#### 2.8.1 RNA extraction

*A. woodii* cells were collected by centrifugation at 7,000 g for 5 min at 4°C, and the pellet was resuspended in 2 mL of RNAprotect (QIAGEN). After a 5 min incubation at room temperature, the cells were collected again, and the pellet was stored at −80°C.

RNA extraction was carried out using a FastRNA Pro Blue kit (MP Bio). Cells were homogenized at 6,400 rpm for 45 s. All the centrifugation steps were carried out for 15 min at 4°C. The RNA was precipitated for 2 h at −80°C, and the RNA pellet was dried for 30 min at room temperature and resuspended in 50 µL of DEPC-H_2_O.

To reduce genomic DNA contamination, samples were digested by TURBO DNase (Ambion). The resulting RNA was purified using the RNeasy Mini kit (QIAGEN). 350 μL of RLT buffer containing β-mercaptoethanol and 200 µL of absolute ethanol were added to the digested samples before purification according to the manufacturer’s instructions.

To check for genomic DNA contamination, a PCR (35 cycles, 50°C, and 2:00 min extension) with primers gDNA_FW and gDNA_RV (Supplementary Table S8), amplifying a 1,997 bp fragment in the *A. woodii pyrE* region, was carried out with Taq DNA polymerase (NEB). When genomic DNA was present, the samples were treated with TURBO DNase and purified with the RNeasy Mini kit a second time. The RNA samples were stored at −80°C.

The RNA concentration was assessed using a Nanodrop Lite spectrophotometer (Thermo Fisher). The quality was evaluated using an Agilent RNA 6000 Nano kit on an Agilent 2100 Bioanalyzer, and the RNA integrity number (RIN) was recorded for all the samples.

#### 2.8.2 Quantitative reverse transcription real-time PCR (RT-qPCR)

Complementary DNA was synthesized from 1 µg of RNA using the Omniscript RT Kit (QIAGEN), random hexamer (Thermo Fisher), and RNase inhibitor (NEB) following the manufacturer’s instructions.

Power SYBR Green (Thermo Fisher) was used for the qPCR, and primer pairs targeting the *gyrA* gyrase gene in the *A. woodii* genome, *orfB* of the plasmid backbones, and the FAST gene employed are listed in Supplementary Table S8. Standard curves allowed for efficiencies of primer pairs to be calculated. Gene expression was calculated for each sample using the Pfaffl method without a calibrator gene:

$$Expression=\frac{{E_{target}}^{-{Cp}_{target}}}{{E_{reference}}^{-{Cp}_{reference}}}.$$

## 3 Results

### 3.1 Automated pipeline for *in silico* analysis of endogenous CRISPR systems

#### 3.1.1 Python script development

A Python script was developed to collect the host organism genome identifier, along with the information found on the CRISPRFinder database (Grissa et al., 2007b; Couvin et al., 2018), and compile all the information collected on each match for each spacer. A schematic overview is presented in Supplementary Figure S1, and a complete diagram is available in Supplementary Figure S2. Briefly, each spacer is run through BLAST and then filtered to reduce the number of false-positives and retain biologically relevant hits.

False-positives were eliminated by removing hits with more than 20% mismatch between spacers and hits, and only one mismatch was allowed in the seven nucleotides seed region adjacent to the PAM. Biologically relevant hits are then used to compile the PAM consensus sequence, based on filtering capabilities for mobile genetic element (MGE) sequences. Hits are kept based on three elements: the title of the hit containing the keywords “phage” or “plasmid;” their presence in a phage sequence predicted by PHASTER; and the annotation of the hit containing the keywords “transposase,” “phage,” “integrase,” or “terminase.”

#### 3.1.2 Evaluation of Python script performance against published literature

The performance of the script was assessed using data available in the literature on Type I-B systems from *Clostridium* spp. that have been successfully used for genome editing. The results are presented in Supplementary Table S9. The script successfully identified the majority of the functional PAMs. For *C. tyrobutyricum,* it failed to identify the TCG alternative (Zhang et al., 2018), and for *C. thermocellum,* it identified only TTA/TTG/TCA instead of TTN and TNA, which have been experimentally validated as functional (Walker et al., 2020). For a majority of those organisms, the script predicted PAMs that had not been identified by manual analysis in the literature (marked with asterisks in Supplementary Table S9). *In vivo* tests are necessary to determine if those PAMs allow for interference and to conclude on the performance of the script.

#### 3.1.3 Analyses of other *Clostridium* spp.

*Clostridium* spp. of interest containing a Type I-B system, *Clostridium butyricum*, *Clostridium limosum*, and *Clostridium novyi* were analyzed using the script. The results are presented in Supplementary Table S10. The results obtained indicate that PAMs containing the nucleotide pairs TC or TT are common in Type I-B CRISPR/Cas systems. The number of candidate PAMs for each organism is quite high, indicating that those CRISPR/Cas systems are likely to recognize multiple PAMs (Fischer et al., 2012).

#### 3.1.4 Proof-of-concept: analysis of the *A. woodii* Type I-B CRISPR system

**FIGURE 1:**
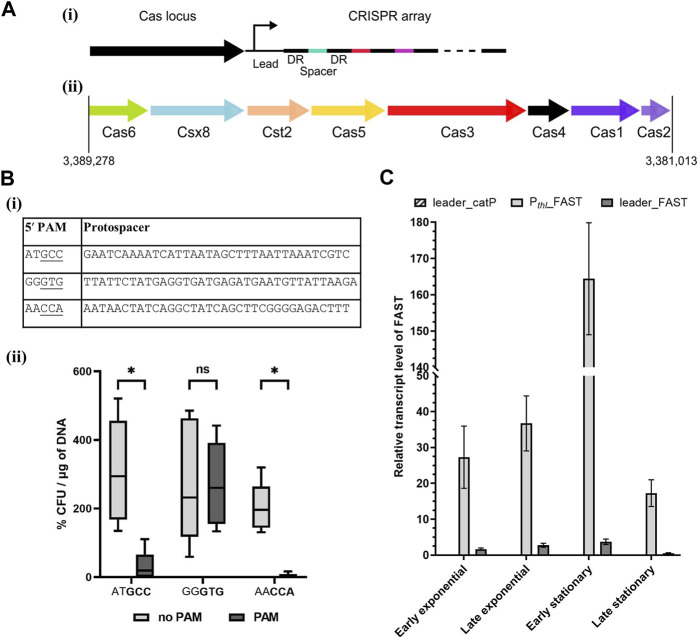
*Acetobacterium woodii* DSM1030 Type I-B CRISPR system. (**A)** Organization of the Type I-B CRISPR system of *A. woodii* DSM1030. (i) Cas locus and CRISPR array relative positions. Both the Cas locus and CRISPR array are in reverse direction in the genome, and the CRISPR array is directly downstream of the Cas locus. (ii) Gene organization of the Cas locus. **(B)** Interference assay to determine functional PAMs in *A. woodii*. (i) Table of the protospacers and PAM candidates used in the interference assay in *A. woodii*. Three of the protospacers identified in Supplementary Table S11 were inserted in a backbone with, in the 5′ position, either the native five nucleotides from the direct repeat “CAAAT” or candidate PAMs. The native 3′ sequence from the direct repeat “ATTTA” was used. Potential functional trinucleotide PAMs are underlined. (ii) Transformation efficiency of WT *A. woodii* obtained with the plasmids containing the different combinations of the protospacer, with (dark gray) or without (light gray) their corresponding candidate PAMs (bold). Data normalized and expressed in percentages with the following parameters: transformation efficiency obtained with the empty backbone control pMTL82151 set to 100% and smallest value of the replicate as 0%. *n* = 5; the 5th and 95th percentile are represented; two-way ANOVA test performed to test for significance: ns: non-significant, *: *p* < 0.033. **(C)** FAST transcript level normalized with *orfB* as the reference gene measured by RT-qPCR at different stages of cell growth. Samples were collected in early and late exponential stages and early and late stationary stages for the pMTL-MPD30 plasmid (dark gray), the negative control pMTL-MPD29 (dashed), and positive control pMTL8415_P_*thl*__FAST (light gray). The transcript levels were calculated with the Pfaffl method without a calibrator sample. Error bars represent technical triplicates.

The CRISPRFinder database (Grissa et al., 2007b; Couvin et al., 2018) identified a Type I-B system in *A. woodii,* as presented in Figure 1A. This system contains a Csx8, a Cas8a homolog, confirmed by protein BLAST with homology to multiple Cas8a proteins from *Clostridium* spp. Cas8a is specific to Type I-A systems, but the gene organization of the *A. woodii* Cas locus is characteristic to Type I-B systems (Makarova and Koonin, 2015). Only one CRISPR array, named array 2, with an evidence level of 4 is identified in the database. It is located directly downstream of the Cas locus; it contains 47 spacers and its direct repeat is “ATT​TAC​ATT​CCA​ATA​TGG​ATC​TAC​TCA​AAT.” This CRISPR array was analyzed using the Python script, and the results are compiled in Supplementary Table S11.

The script identified multiple matches with invading elements but none with less than 17% mismatch. Although the script computed no clear consensus sequence for the 5′ PAM, three out of the eight matches contain two consecutive cytosines. Interestingly, a match with a 5′ CCA PAM in a phage gene was found, and this PAM was shown to be functional for the *C. difficile* Type I-B system (Boudry et al., 2015; Maikova et al., 2019). Thus, it is the main candidate for harnessing the endogenous CRISPR system in *A. woodii*.

### 3.2 Harnessing of the *A. woodii* endogenous CRISPR system

#### 3.2.1 PAM validation by interference assay

Interference assays are used to assess PAM functionality by transformation of the WT strain with plasmids containing a protospacer and different candidate PAMs. Functional PAMs trigger interference and result in lower transformation efficiencies (Pyne et al., 2016; Walker et al., 2020). Three candidate 5′ PAMs identified by the Python script were tested experimentally in *A. woodii*, as shown in Figure 1Bi. Mismatches adjacent to the PAM reduce the likelihood of effective interference. Therefore, protospacers with no mismatch in the first position and with only one mismatch in the first 10 nucleotides were selected for testing. Each protospacer was assembled into a plasmid with the 3′ sequence from the direct repeat “ATTTA” and either the 5′ sequence of the direct repeat “CAAAT” or its corresponding PAM candidate. WT *A. woodii* was separately transformed with the six plasmids, pMTL-MPD1 to 6; transformation efficiencies were expressed as a percentage of the transformation efficiency obtained with the pMTL82151 positive control and normalized to the highest and lowest values.

Although high variations were observed between replicates (Figure 1Bii), the reduction in transformation efficiency in the presence of the GCC and CCA PAMs was shown to be statistically significant (*p-*value <0.033) compared to the presence of the protospacers alone. Sanger sequencing of colony PCR amplicons showed a high prevalence of mutations in the CCA PAM, with one of the cytosines missing. Those colonies with mutated PAMs are thus escape mutants of the endogenous CRISPR/Cas system. Taken together, these results strongly suggest that the double cytosine is essential to the interference phase of the studied Type I-B CRISPR/Cas system. The CCA 5′ PAM was selected for subsequent genome editing experiments in *A. woodii*.

#### 3.2.2 Leader sequence characterization

For efficient targeting of invading DNA, the CRISPR array needs to be transcribed into pre-crRNA before maturation and Cascade assembly. In order to harness the *A. woodii* CRISPR system, a better characterization of the leader sequence responsible for the transcription of the array is required. For that purpose, the plasmid pMTL-MPD30 was built with the FAST reporter gene placed downstream of the leader of the aforementioned array 2. Control plasmids were built with either the *catP* reporter gene replacing the FAST reporter gene downstream of the leader sequence or with the thiolase promoter replacing the leader sequence for expression of the FAST-encoding reporter gene. These controls confirm primer specificity and efficiency, respectively. The transcript levels of the FAST gene were expressed (Figure 1C) with *orfB* as the reference gene to take into account the plasmid copy number. Transcript levels of the controls confirmed adequate annealing and specificity of the primers. The leader sequence allows for a low level of transcription in all cell growth stages with a clear reduction in the RNA level in the late stationary phase. This experiment confirms the functionality of the leader sequence for expression of CRISPR arrays for targeting DNA.

#### 3.2.3 Genome engineering capability validation: *pyrE* knock-out

**FIGURE 2:**
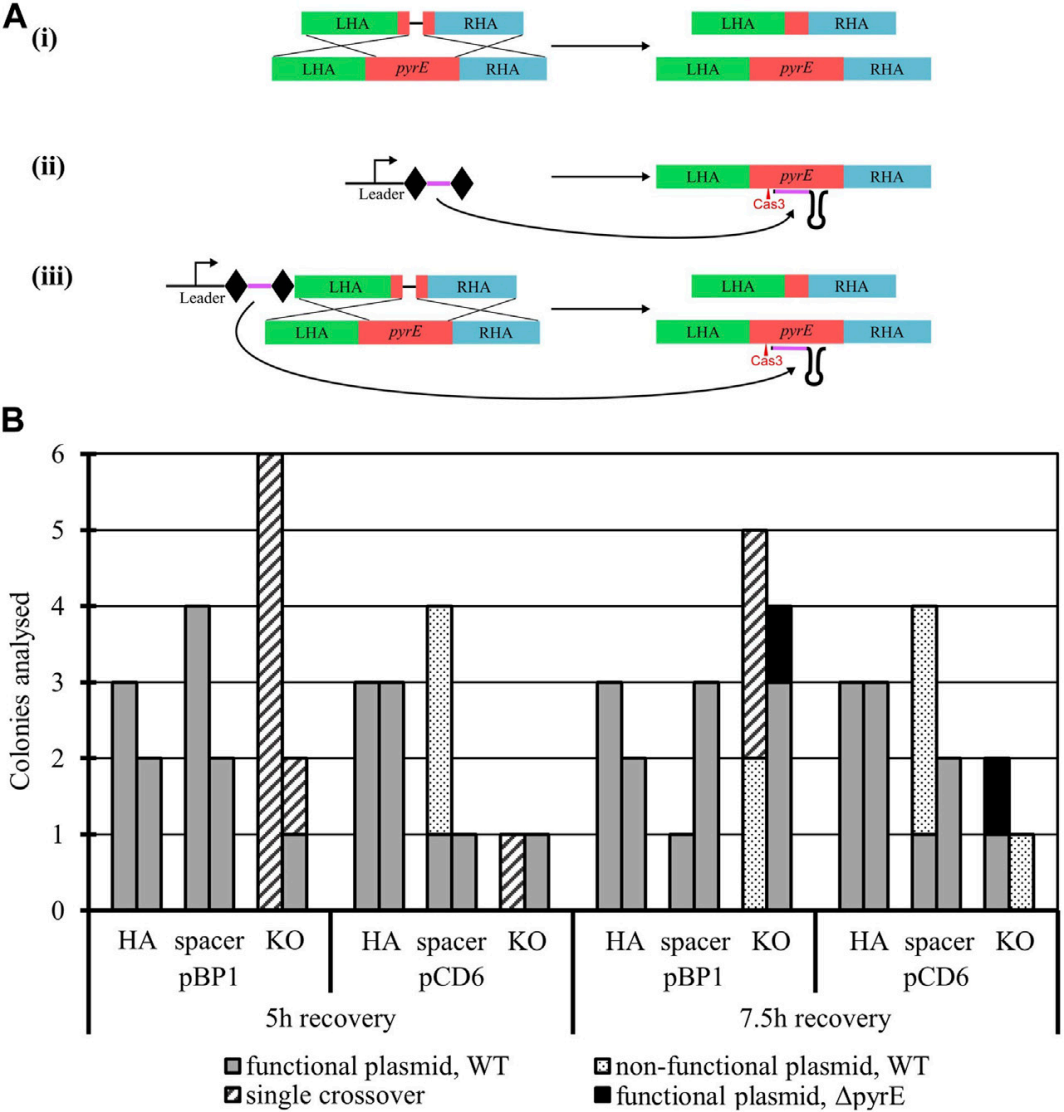
Endogenous CRISPR-based deletion of the *A. woodii pyrE* gene. **(A)** *pyrE* deletion strategy in *A. woodii* using its native Type I-B CRISPR system. The constructs represented correspond to the application-specific module of the following plasmids, each of them built with either pBP1 or pCD6 as the Gram-positive replicon: (i) pMTL-MPD9 and 12 HAs alone (471 bp LHA in green; 491 bp RHA in blue) allow for homologous recombination in some of the cells of the population; (ii) pMTL-MPD10 and 13, the expression of the 36 bp spacer (purple) flanked by DRs (diamonds) alone leads to self-cleavage by Cas3 (red) at the *pyrE* gene (red) and cell death; (iii) pMTL-MPD11 and 14, the full editing cassette allows for homologous recombination, deletion of the 300 bp region, and selection of the successful KO. **(B)** Summary of colonies analyzed for the 300 bp deletion in the *pyrE* gene by colony PCRs with primers AW_CRISPR_pyrE_armF and AW_pyrEcomp_RHAR. Data show two replicates of transformation assays with pBP1 and pCD6 Gram-positive replicon for recovery times of 5 h and 7.5 h. Where possible, up to six colonies were analyzed. Gray: WT *pyrE* locus with functional corresponding plasmid; dots: WT *pyrE* locus with non-functional corresponding plasmid; dashed: single crossover of the plasmid in the *pyrE* locus; black: Δ*pyrE* with the functional corresponding plasmid.

To establish the potential of the *A. woodii* Type I-B CRISPR/Cas system for genome editing, the *pyrE* gene was targeted for inactivation through the creation of a 300 bp deletion known to lead to uracil auxotrophy (Baker et al., 2022). Six plasmids were constructed (Figure 2A) with HAs alone, spacer alone, or the whole editing cassette and either the pCD6 or pBP1 Gram-positive replicon. The vectors with HAs and spacer alone function as controls to assess the impact of homologous recombination and self-targeting by Cas3, respectively. Only the whole editing cassette with HAs and expressed spacer allows for homologous recombination and selection against WT by self-targeting. WT *A. woodii* was separately transformed with the aforementioned plasmids in biological duplicates and plated after 5 h and 7.5 h of recovery.

The presence of the expressed spacer targeting the *pyrE* gene, with either replicon pCD6 or pBP1, with or without HAs, significantly reduced transformation efficiencies to between 0.06% and 2.17% of the transformation efficiency obtained with the corresponding plasmid containing HAs alone (data not shown). This shows the efficient self-targeting by the endogenous CRISPR/Cas system of the *pyrE* gene, leading to cell death. Colony numbers obtained varied significantly between the different replicates; colony PCR results are presented in Figure 2B. Successful gene knock-out was obtained with the full editing cassette, with either Gram-positive replicon pBP1 or pCD6. The latter was found to lead to 100% plasmid loss after one subculture without selection (Baker et al., 2022); it was, therefore, chosen for subsequent editing vectors.

Analysis of the escape mutants revealed single crossover events and frequent deletion of the spacer. The deletion of the spacer can be explained by the presence of the identical direct repeats flanking the spacer, facilitating recombination under Cas3 selective pressure.

#### 3.2.4 Impact of homology arm length on editing efficiency: *pheA* knock-out

The impact of homology arm length on editing efficiency was studied, tested with 0.5 kb, 1.0 kb, and 1.5 kb, for a 354 bp in-frame deletion in the *pheA* gene, previously shown to lead to phenylalanine auxotrophy (Baker et al., 2022).

**FIGURE 3:**
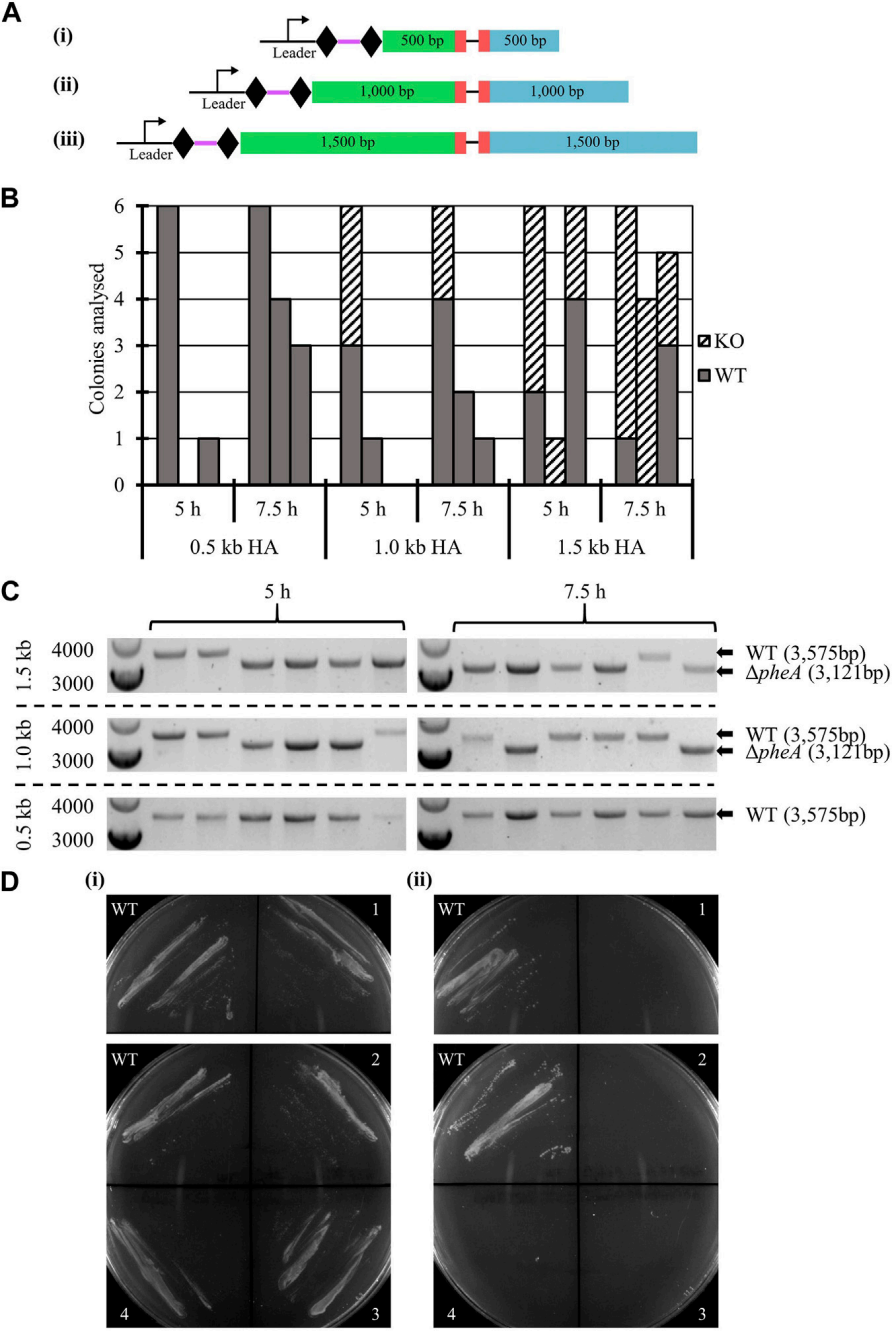
Endogenous CRISPR-based deletion of the *A. woodii pheA* gene. **(A)** Representation of the application-specific modules of the three different editing plasmids containing different size HAs for *pheA* (red) KO in *A. woodii*. The 35 bp spacer (purple) is flanked by the native DR (diamond) and expressed under the control of the native leader sequence. HAs (LHA in green; RHA in blue) of different lengths allow for homologous recombination and deletion of the 354 bp region: (i) pMTL-MPD19, (ii) pMTL-MPD20, and (iii) pMTL-MPD21. **(B)** Summary of colonies analyzed for the 354 bp deletion in the *pheA* gene by colony PCRs with primers FW_pheAHA1.5_out and RV_pheAHA1.5_out. Data show three replicates of transformation assays with homology arms of 0.5 kb, 1.0 kb, and 1.5 kb for recovery times of 5 h and 7.5 h. Where possible, up to six colonies were analyzed; missing bars show conditions where no colonies were obtained. Gray: WT *pheA* locus; dashed: Δ*pheA*. **(C)** Colony PCR results for the first replicate of the *pheA* KO experiment with homology arms of 0.5 kb, 1.0 kb, or 1.5 kb by amplification with primers FW_pheAHA1.5_out and RV_pheAHA1.5_out. The WT amplicon is expected at 3,575 bp and the KO amplicon at 3,121 bp. **(D)** Phenotypic analysis of the *A. woodii* Δ*pheA* strains obtained. 1 and 2 are *A. woodii* Δ*pheA* strains obtained with 1.0 kb HAs with 5 h or 7.5 h recovery time, respectively; 3 and 4 are *A. woodii* Δ*pheA* strains obtained with 1.5 kb HAs with 5 h or 7.5 h recovery time, respectively; WT is the WT *A. woodii* strain. (i) Minimal media supplemented with 20 μg/mL of phenylalanine; (ii) minimal media without supplementation.

WT *A. woodii* was separately transformed with six vectors (Figure 3A), three containing the HAs alone and three editing plasmids also containing the expressed spacer. The transformation efficiency obtained with each editing vector was normalized to the transformation efficiency obtained with the corresponding vector containing only the HAs. While low transformation efficiencies and high variations in colony numbers between each replicate were observed, the presence of the spacer reduced the transformation efficiency to between 0.053% and 0.32% of the efficiency obtained with the vectors carrying HAs alone (data not shown), showing efficient targeting of the *A. woodii* genome by the selected spacer. Characteristics of the colonies analyzed for the three replicates of this experiment are shown in Figures 3B, C, as well as the results of the colony PCRs of the *pheA* locus for the first replicate of this experiment.

At both 5 h and 7.5 h recovery, the 1.5 kb HAs resulted in more consistent knock-out generation with between 33.3% and 100% gene editing efficiency. After the plasmid loss protocol, 91.7% of colonies tested (72 in total) had successfully lost the plasmid. The phenotype of the strains was confirmed by plating on minimal media with and without phenylalanine supplementation (Figure 3D).

These results are the first report of successful gene editing using the *A. woodii* endogenous Type I-B CRISPR/Cas system and represent a promising basis for further engineering of this chassis. The knock-out of *pheA* followed by plasmid loss and confirmation of the phenotype highlights how straightforward and rapid the technique is, while illustrating the importance of the length of the editing template.

#### 3.2.5 Validation of the system: 3.2 kb *hsdR1* knock-out

**FIGURE 4:**
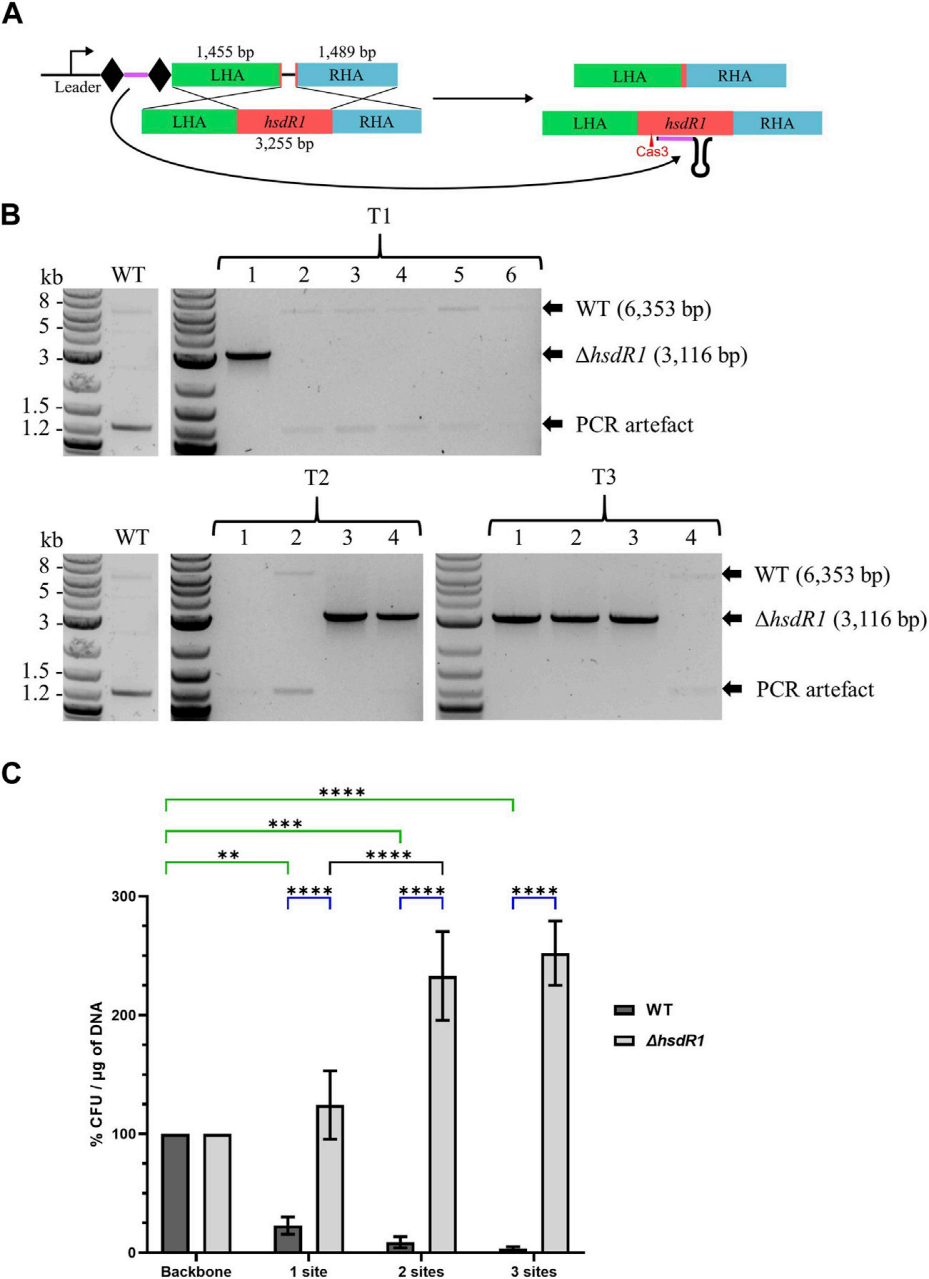
Endogenous CRISPR-based deletion of the *A. woodii hsdR1* gene. **(A)** Representation of the application-specific module of the editing plasmid for *hsdR1* (red) KO in *A. woodii*, pMTL-MPD22. The 36 bp spacer (purple) is flanked by the native DR (diamond) and expressed under the control of the native leader sequence. The editing template with a 1,455 bp LHA (green) and a 1,489 bp RHA (blue) allows for homologous recombination and deletion of a 3,237 bp region. **(B)** Colony PCR results with primers FW_hsdR1_screen_1.5 and RV_hsdR1_screen_1.5 of the *hsdR1* locus of colonies transformed with the editing vector for *hsdR1* KO. The WT amplicon is expected at 6,353 bp, and KO amplicon is expected at 3,116 bp. T1: standard *A. woodii* electroporation; T2: high-efficiency protocol with 100 μL cells and 1 µg of DNA; T3: high-efficiency protocol with 200 μL cells and 4 µg of DNA. **(C)** Transformation efficiencies of *A. woodii* WT and Δ*hsdR1* obtained with plasmids containing up to three R-M sites normalized to the transformation efficiency obtained with the empty backbone pMTL82151. Data represent technical triplicates for WT and biological triplicates for Δ*hsdR1*. Two-way ANOVA tests were performed to test for significance, in green, of the impact of the presence of 1–3 R-M sites compared to the empty backbone on transformation efficiency of WT *A. woodii*; in blue, for confirmation of the phenotype of *A. woodii* Δ*hsdR1* compared to WT for each plasmid. **: *p* = 0.0010; ***: *p* = 0.0002; ****: *p* < 0.0001.

To test the robustness of the system for gene KO in *A. woodii*, the restriction subunit gene *hsdR1* responsible for cleavage of the *cas9*-containing plasmids was targeted. Deletion of this gene has never previously been generated and at 3.2 kb represents a 10-fold increase in the size of the DNA deleted. WT *A. woodii* was transformed with the editing vector pMTL-MPD22 (Figure 4A), following protocols with increasing cell densities and DNA amounts referred to as T1, T2, and T3. Plating after 7.5 h of recovery yielded only WT colonies (data not shown). The generation of knock-out mutants was demonstrated (Figure 4B) with all three methods of transformation plated at 5 h of recovery, with 16.7%, 50%, and 75% efficiency for T1, T2, and T3, respectively. Sanger sequencing of the PCR-amplified plasmid sequences for T1 colonies 1 and 2; all T2 colonies; and T3 colonies 1, 2, and 4 revealed the absence of the spacer in the plasmid of colonies T1 colony 2 and T3 colony 4. As previously observed, recombination between the direct repeats flanking the spacer facilitates the excision of the spacer from the plasmid, rendering it non-functional. All Δ*hsdR1* colony PCR amplicons were confirmed by Sanger sequencing. Plasmid loss was undertaken for the six confirmed Δ*hsdR1* colonies, with 80.6% of those tested (36 in total) being shown to have successfully lost the plasmid.

To confirm the impact of the R-M site on transformation efficiency and the phenotype of the *hsdR1* deletion, the R-M site 5′-TAAGN_5_TCC-3′ was inserted in one, two, or three copies in the backbone pMTL82151. WT and Δ*hsdR1 A. woodii* strains were separately transformed with the four plasmids. For two of the replicates, the transformation efficiency of the Δ*hsdR1* strain with the empty backbone was lower than the transformation efficiency of the WT, representing 20.59% and 52.94% of the WT transformation efficiency. More experiments are needed to determine if this is due to the *hsdR1* deletion or to competent cell preparation variations.

As shown by the two-way ANOVA test presented in green in Figure 4C, the presence of the R-M site on plasmids has a significant impact on the transformation efficiency of WT *A. woodii*. Increasing numbers of recognition sites negatively impact the transformation efficiency. Deletion of the HsdR1 subunit of the R-M system significantly increased relative transformation efficiencies obtained with plasmids containing the R-M recognition site compared to WT, as shown by the two-way ANOVA test shown in blue. This confirms recognition of the 5′-TAAGN_5_TCC-3′ motif by the HsdR1 subunit. Surprisingly, the presence of two or three R-M sites on the plasmids resulted in an increased relative transformation efficiency of *A. woodii* Δ*hsdR1* compared to plasmids with none or one site.

#### 3.2.6 Validation of the system: reporter gene knock-in at the *pheA* locus

**FIGURE 5:**
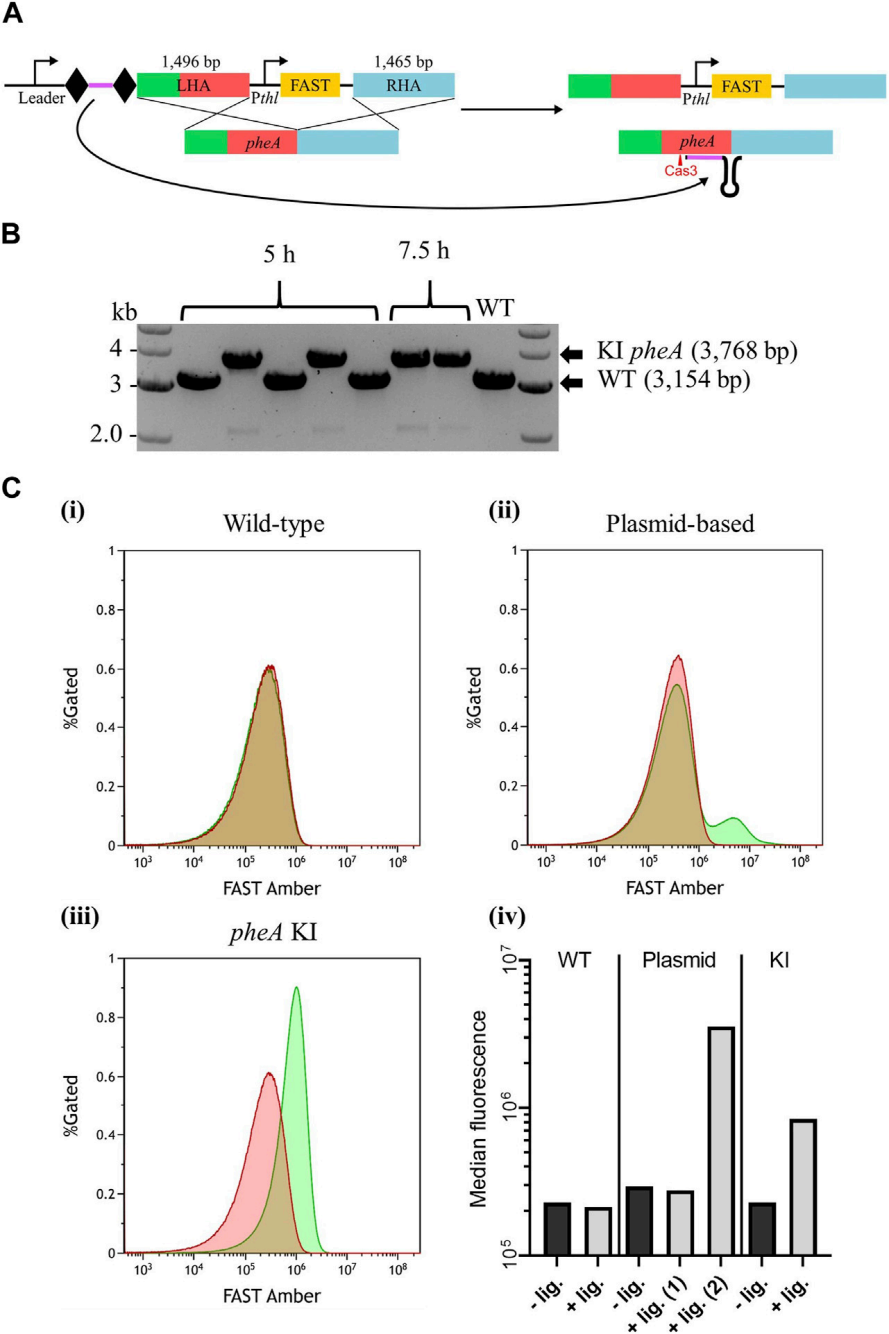
Endogenous CRISPR-based insertion of P_*thl*__FAST at the *pheA* locus in the *A. woodii* genome. **(A)** Representation of the application-specific module of the editing plasmid for P_*thl*__FAST insertion at the *pheA* (red) locus in *A. woodii*, pMTL-MPD24. The 36 bp spacer (purple) is flanked by the native DR (diamond) and expressed under the control of the native leader sequence. The editing template with a 1,496 bp LHA (containing *pheA*) and a 1,465 bp RHA (blue) allows for homologous recombination and insertion of the cargo. **(B)** Colony PCR results with primers FW_pheA_KI_seq and RV_pheA_KI_seq of the *pheA* locus of colonies transformed with the editing vector. The WT amplicon is expected at 3,154 bp, and the KI amplicon is expected at 3,768 bp. **(C)** Flow cytometry results for (i) *A. woodii* WT, (ii) *A. woodii* WT with pMTL8415_P_*thl*__FAST, and (iii) *A. woodii* P_*thl*__FAST *pheA* KI. Cell populations with PBS only (red) or with the ^TF^Amber ligand (green), and the percentage of cells gated is represented as a function of the FAST Amber fluorescence intensity. The number of events represented is >440,000 for WT and >1,200,000 for all other strains; and exact numbers can be found in Materials and Methods. (iv) Median fluorescence of the populations with PBS only (dark gray) or with the ^TF^Amber ligand (light gray). For the plasmid-based expression strain, the median fluorescence was calculated separately for the two peaks observed and annotated (1) and (2).

As a final test to the robustness of *A. woodii* Type I-B CRISPR/Cas system as a genetic engineering tool, its capacity to knock-in a reporter gene was tested. As no 5′ CCA trinucleotide could be found in the target intergenic region downstream of *pheA*, an alternative 5′ CCG PAM was chosen for spacer design. Its selection was based on the aforementioned interference assay, suggesting that the double cytosine alone is essential for efficient targeting. WT *A. woodii* was transformed with the editing vector pMTL-MPD24 (Figure 5A), which was designed to insert a 614 bp DNA fragment expressing the FAST reporter gene downstream of *pheA* in place of the targeted PAM.

Based on the PCR analysis of six colonies from plating at 5 h and 7.5 h, successful knock-ins were obtained with both recovery times (Figure 5B), with an overall efficiency of approximately 57%. Following plasmid loss, plating of the clones onto minimal media with and without supplementary phenylalanine confirmed that the insertion had not disrupted *pheA* (Supplementary Figure S3). Expression of FAST was assayed using flow cytometry (Figure 5C), with WT *A. woodii* and WT *A. woodii* with P_*thl*__FAST in a pMTL84151 plasmid being used as negative and positive controls, respectively*.* As expected, WT *A. woodii* displayed the same level of fluorescence with and without the ligand. In the presence of the ligand, the plasmid-based population fluorescence was heterogeneous with two clear cell populations; one population displayed a level of fluorescence similar to the level of fluorescence without a ligand and a smaller one displayed a higher level of fluorescence. The KI strain, on the contrary, displayed a homogeneous population with a level of fluorescence higher than that without the ligand. It is important to note that the fluorescence of the KI population (KI + ligand in Figure 5Civ) is lower than the fluorescence of the plasmid-based fluorescent population (plasmid + ligand (2) in Figure 5Civ). The auto-fluorescence of the plasmid-based population is also slightly higher than that of both the WT and KI strains.

### 3.3 Proof-of-concept: harnessing the endogenous CRISPR system of *Clostridium autoethanogenum*

#### 3.3.1 Description of the CRISPR locus in *Clostridium autoethanogenum* and PAM prediction with Python script

In *C. autoethanogenum*, the CRISPRFinder database identified a Type I-B system between positions 1,491,001 and 1,499,580 in reverse orientation. The database did not predict a Cas8 gene, which is essential for a functional CRISPR system. A protein BLAST of the gene situated between the Cas7 and the Cas6 revealed homologies to proteins labeled Cas8b in other *Clostridium* spp. For instance, an alignment with the Cas8b protein of the Type I-B system of *Clostridium tetani* has an E-value of 1e-148. Overall, the gene organization is the same as the one described for *A. woodii.*

Four CRISPR arrays were identified, including three with an evidence level of 4; array 2 is directly downstream of the Cas locus with 21 spacers, while array 3 (42 spacers) and array 4 (33 spacers) are immediately upstream of the Cas locus. All these arrays have a similar direct repeat, with only one nucleotide varying: “ATTTAAATACATCT(C/T)ATGTTGAGGTTCAAC.” This suggests that they are related and should yield coherent results when run in the Python script. Hits with less than 15% mismatch are presented in Supplementary Table S12 (hits with mismatch between 15% and 20% are presented in Supplementary Table S13 for further information). Array 4 had the most hits with 15 in phage regions or phage-related genes.

Overall, the Python script predicted a TCH/TTR putative 5′ PAM. Most notably, the spacer in position 27 of this array is a perfect match to a phage-related gene in *Clostridium ljungdahlii* with, in position 5′, the sequence “CTTCA.” The 5′ TCA PAM was selected for harnessing the endogenous system as a genetic engineering tool in *C. autoethanogenum*.

#### 3.3.2 Genome engineering capacity validation by *pyrE* knock-out in *C. autoethanogenum*

**FIGURE 6:**
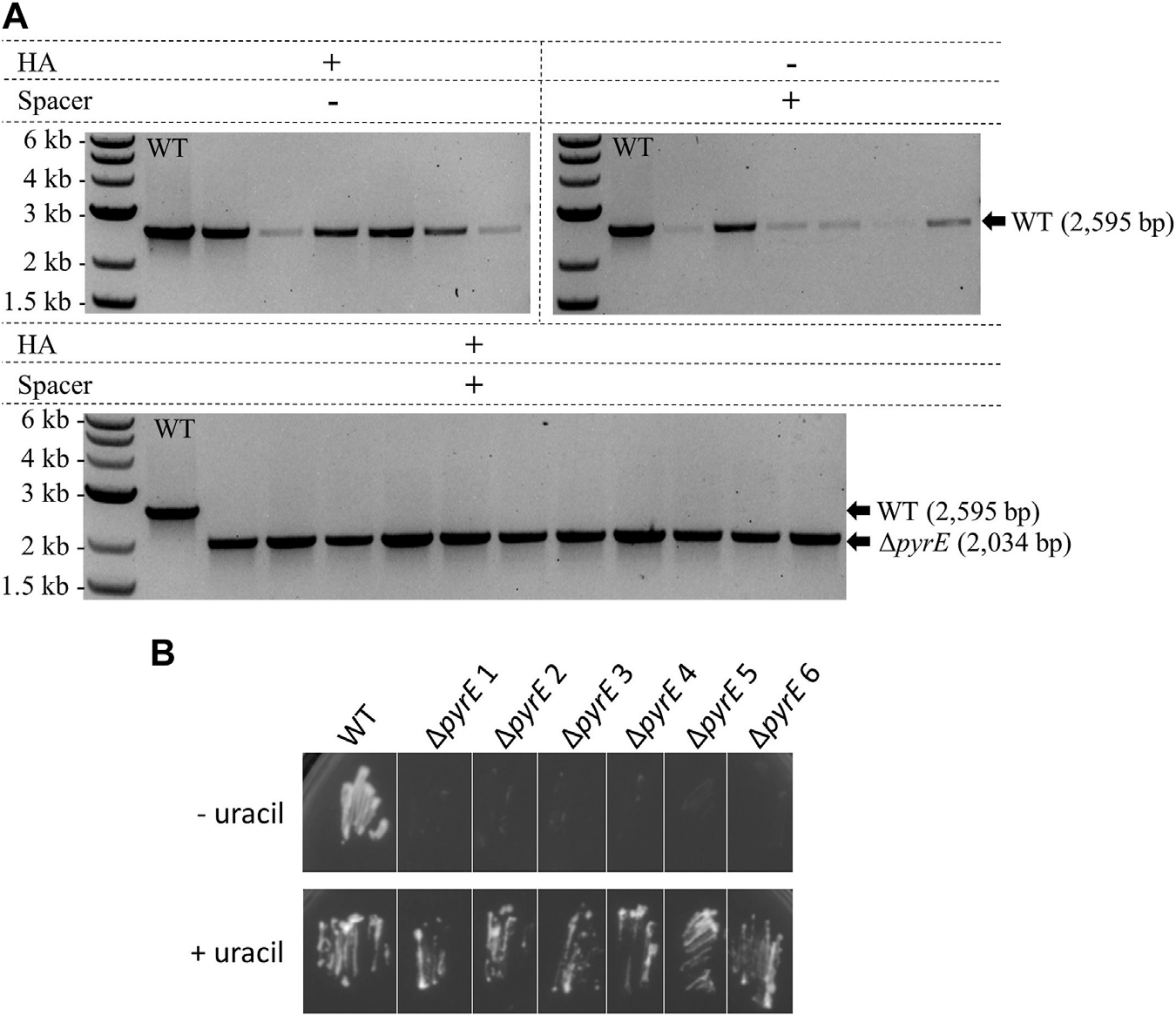
Endogenous CRISPR-based deletion of the *C. autoethanogenum pyrE* gene. **(A)** Colony PCR results for the *pyrE* locus of *C. autoethanogenum* with primers FW_pyrECLAU_screening and RV_pyrECLAU_screening; the WT amplicon is expected at 2,595 bp and the KO amplicon at 2,034 bp. + and —indicate the presence or absence of the corresponding element, respectively. **(B)** Phenotypic analysis of the *C. autoethanogenum* Δ*pyrE* strains obtained. WT and six colonies of Δ*pyrE C. autoethanogenum* strains obtained after plasmid loss were plated on minimal media supplemented (+ uracil) or not (- uracil) with 20 μg/mL of uracil.

Design of the *pyrE* KO editing vector was based both on a 561 bp in-frame *pyrE* deletion already performed in *C. autoethanogenum* in our laboratory with CRISPR/Cas9 and on its native CRISPR array 4. WT *C. autoethanogenum* was conjugated with three plasmids containing HAs alone, expressed spacer alone, and both HAs and expressed spacer, as described for *A. woodii* in Figure 2A. Colony PCR analysis of transformants (Figure 6A) demonstrated that the editing vector containing both the HAs and the expressed spacer resulted in an editing efficiency of 100%. In contrast, vectors harboring only one part of the editing cassette did not result in any Δ*pyrE* mutants. Plasmid loss was achieved; the additional subculture without selection as described in Materials and Methods increased the plasmid loss rate from 33.3% to 58.3% (out of 12 colonies tested in both conditions). The phenotype of the strains obtained after plasmid loss was confirmed by plating on minimal media with and without uracil supplementation (Figure 6B). This is the first report of the successful use of the *C. autoethanogenum* Type I-B endogenous system for genome editing and demonstrates the robustness of the developed workflow—from the automated PAM identification pipeline to gene knock-out.

## 4 Discussion

### 4.1 *In silico* analyses

Endogenous CRISPR/Cas systems offer alternative genome editing tools for microorganisms that contain them. They have, in some cases, proven to be superior to using the highly toxic *S. pyogenes* Cas9 (Pyne et al., 2016; Zhou et al., 2021) and have enabled genome editing of otherwise genetically recalcitrant bacteria (Hidalgo-Cantabrana et al., 2019). PAM identification is the first necessary step. *In silico* strategies are widely employed (Zhou et al., 2011; Arndt et al., 2019), requiring the analysis of considerable volumes of data to derive an appropriate catalog of potential PAM candidates. A variant of that strategy is the use of CRISPRTarget, a tool created to predict the targets of CRISPR RNAs (Biswas et al., 2013; Biswas et al., 2015) and to characterize the exposure history of the strains (Zheng et al., 2019; Xu et al., 2020). *In vivo* techniques are also employed to identify PAMs such as plasmid depletion assays (Walker et al., 2020) or PAM-SCANR (Leenay et al., 2016). These require extensive library construction or cloning and rely on large-scale sequencing. Accordingly, PAM identification is recognized as one of the challenges of exploiting endogenous CRISPR systems for genetic engineering (Zhou et al., 2021).

In this study, a dedicated bioinformatics tool was successfully developed to allow fully automated predictions based on data from the CRISPRFinder database. In the meantime, two other tools to identify PAMs have been published (Vink et al., 2021; Rybnicky et al., 2022). In all three tools, spacers are run through BLAST, and the flanking sequences of the matches are retrieved to compile a prediction of the PAM. The main advancement made over preceding tools is filtering of hits. False-positives are filtered based either on global mismatch (Vink et al., 2021) or a combination of parameters, including gap number, e-value, length of the hit, and starting position of the hit (Rybnicky et al., 2022). Our Python script filters on global mismatch but is more stringent in the seed region adjacent to the PAM, as interference relies on the recognition of a seed region (Semenova et al., 2011; Xue et al., 2015). As CRISPR/Cas systems are prokaryotic defense mechanisms against invading elements (Makarova and Koonin, 2015), filtering of biologically relevant hits relies on their presence in mobile genetic elements (MGEs), including plasmids, phages, prophages, and any related genes. The similar performances between Spacer2PAM (Rybnicky et al., 2022) and our Python script, despite a more comprehensive filtering parameter selection for false-positives in Spacer2PAM, could be explained by our refined selection for biologically relevant hit sequences.

As *C. autoethanogenum* is an important industrial strain, its CRISPR/Cas system has been of interest in recent years (Pyne et al., 2016; Rybnicky et al., 2022) and can serve as a case study to compare the different methods of PAM elucidation. The output of our Python script was two putative 5′ PAMs, TCH and TTR, and TCA was confirmed *in vivo* by genome editing. In previously performed manual PAM prediction (Pyne et al., 2016), the array direction was incorrect, leading to the 3′ PAM being wrongly selected as the potential PAM. Spacer2PAM only predicted a TTNN PAM consensus, while a NYCN consensus was experimentally confirmed (Rybnicky et al., 2022). Lastly, Vink et al. (2021). showed a direct link between the direct repeat sequence and the identified PAMs. Spacers from different microorganisms with identical direct repeats were clustered and PAM prediction achieved for each cluster. The PAM prediction corresponding to *C. autoethanogenum* direct repeats was TCNX, corroborated by *in vivo* experiments performed in this study and by Rybnicky et al. (2022).

For the *A. woodii* Type I-B CRISPR system, the Python script did not manage to compute a clear consensus, but allowed the rapid identification of matches with MGEs and their corresponding PAM candidates. *In vivo* experiments subsequently confirmed the functionality of one of these PAMs.

CRISPR systems have been shown to recognize a variety of PAMs. Mismatched PAMs can lead to interference or primed adaptation depending on the spacer analyzed (Xue et al., 2015), and it is hypothesized that a wider variety of PAMs can be recognized for interference compared to adaptation. This would result in a better defense system for the host microorganism, which would be less likely to suffer from evasion from mutated MGEs or closely related MGEs (Xue et al., 2015; Rybnicky et al., 2022). This diversity of functional PAMs is proving to be challenging for both *in silico* prediction and *in vivo* validation and could explain the complex results obtained from the Python script for some microorganisms. Conveniently, Spacer2PAM (Rybnicky et al., 2022) provides a PAM score to help the user judge the quality of each PAM within one CRISPR/Cas system. Future tools could use that score but also optimize the output to reflect the functional PAM diversity without compromising user-friendliness and clarity.

A better understanding of the adaptation and the interference mechanisms is necessary to adapt the parameters for PAM identification, but all of those recently developed tools and their outputs pave the way for a PAM prediction pipeline combining (1) spacer BLAST to find MGE matches and (2) direct repeat homology to find candidate PAMs based on both *de novo* identification and mining of published PAMs.

### 4.2 Proof-of-concept for the use of the endogenous CRISPR/Cas system for genome engineering in *A. woodii* and *C. autoethanogenum*

In this study, proof-of-concept for the complete workflow of genome editing with the endogenous CRISPR/Cas system was established in *A. woodii*. After *in silico* analyses, the PAM was confirmed *in vivo* and the leader sequence was characterized. Similar to the interference assay completed by Rybnicky et al. (2022), the transformation efficiencies observed were variable between the five replicates. However, the assay showed that the double cytosine is essential for interference in *A. woodii,* and this result was confirmed by successful genome engineering experiments with both CCA and CCG PAMs.

When using the endogenous CRISPR system, providing homology arms for homologous recombination allows evasion from self-targeting and cell death. Other pathways exist for CRISPR-based targeting evasion, including disruption of targeting by mutation of either the target site or the targeting mechanisms (Wimmer and Beisel, 2019). Analysis of the escape mutants obtained in our study predominantly revealed inactivation of the CRISPR-based targeting by loss of the plasmid-encoded spacer, likely through recombination of the direct repeats (Gomaa et al., 2014; Canez et al., 2019). Consequently, optimizing DNA transfer and homologous recombination is essential; different parameters are reported to have an impact on editing efficiency, including spacer design and expression, Gram-positive replicon, editing template length, and cell density; they were each investigated with successive deletion targets and are discussed below.

In *A. woodii* and *C. autoethanogenum*, the spacers were chosen to have lengths corresponding to the average length of the protospacers in the native arrays, and a GC content of between 40% and 60% (Javaid and Choi, 2021; Zhou et al., 2021). Other parameters for spacer design appear to have an impact on interference efficiency but remain elusive. In bacteria, the spacer sequence was shown to have an impact on interference (Xue et al., 2015; Bourgeois et al., 2020). Studies performed in mammalian cells with CRISPR/Cas9 have demonstrated that the sgRNA sequence impacts interference, likely through sgRNA transcription and stability (Doench et al., 2014; Wu et al., 2014). Target sequence accessibility also modulates interference efficiency (Wu et al., 2014; Chari et al., 2015).

Spacer expression replicating the native arrays was effective in *A. woodii* and *C. autoethanogenum,* as was also found in previous studies (Pyne et al., 2016; Hidalgo-Cantabrana et al., 2019; Zheng et al., 2019). Other studies have found that expression with the leader sequence can be inadequate (high or low), leading either to cell death, potentially because spacer expression happens before successful double crossover occurs (Maikova et al., 2019; Zhou et al., 2021), or to inefficient genome editing (Walker et al., 2020; Xu et al., 2020). In these cases, genome editing was successfully achieved by expression of the array with an inducible promoter or a strong constitutive promoter, respectively.

The initial generation of *pyrE* KO in *A. woodii* was successful using both the pCD6 and pBP1 replicons. Subsequent editing experiments were confined to plasmids based on the former replicon in view of its comparative instability (Baker et al., 2022), a desirable property when plasmid loss is needed. Targeting of *pheA* confirmed that the use of editing templates comprising larger HAs (1.5 kb compared to 0.5 kb and 1.0 kb) has a positive impact on genome editing efficiency (Zheng et al., 2019). Not unexpectedly, editing efficiencies were also enhanced through the use of more effective transformation procedures. In *C. autoethanogenum*, Gram-positive replicon, homology arms, and transformation protocol previously used for CRISPR/Cas9 genome editing led to successful editing with its endogenous CRISPR system.

As expected, deletion of the *hsdR1* gene in *A. woodii* resulted in an increased relative frequency of transfer of plasmids carrying its corresponding restriction recognition site(s). Interestingly, plasmids containing two or three R-M sites resulted in slightly higher transformation efficiencies of the *A. woodii* Δ*hsdR1* strain compared to the backbone alone or with just one R-M site. This suggests that the methylation and specificity subunits of the system confer an advantage for transformation of *A. woodii* with plasmids containing the R-M recognition site in the absence of the restriction subunit. In *E. coli* K-12, a point mutation in the restriction subunit resulted in an r^-^m* strain with enhanced modification (Kelleher et al., 1991). Adenine-specific methylation systems have complex and diverse functions in bacterial cells, including, but not limited to, regulation of conjugation, DNA replication, and various transcriptional and posttranscriptional gene regulations (Wion and Casadesus, 2006; Low and Casadesus, 2008; Vasu and Nagaraja, 2013; Adhikari and Curtis, 2016). Further study is necessary for a full understanding of the impact of the *hsdR1* deletion in *A. woodii*.

It is interesting to note that when the FAST reporter was present on an autonomous plasmid in *A. woodii,* FAST expression showed a heterogeneous population. Similar studies have observed the same phenomenon in *A. woodii* (Mook et al., 2022), in *C. acetobutylicum* (Streett et al., 2019), and *Eubacterium limosum* (Flaiz et al., 2021). The reason for this heterogeneity is not entirely clear, but it was not observed when the FAST gene was integrated into the genome, indicating that it is a consequence of the reporter gene being localized on an autonomous plasmid. Accordingly, if population heterogeneity is to be avoided in the future, desirable metabolic pathways should be integrated into the genome.

## 5 Conclusion

This study reports the development of a Python script for fast and efficient PAM elucidation for the use of endogenous CRISPR/Cas systems as genome engineering tools. After validation against published literature, it allowed for characterization of Type I-B endogenous systems of two industrially relevant microorganisms for their use as genome engineering tools in only a few steps. Editing cassettes were designed to be synthetic CRISPR arrays, replicating the native arrays of each host microorganism. They consisted of the leader sequence expressing an adequate spacer, flanked by direct repeats. The editing efficiencies varied between targets and between microorganisms, but this work is the first report of the successful use of the endogenous CRISPR/Cas systems of *A. woodii* and *C. autoethanogenum* for genetic engineering.

Compared to most CRISPR/Cas9-based techniques, the workflow presented here represents a major improvement for genome editing tools for recalcitrant microorganisms like *A. woodii* and will allow the leveraging of a widespread range of bacteria containing endogenous CRISPR systems. It removes the need for sophisticated inducible promoter systems necessary to mitigate c*as9* toxicity (Canadas et al., 2019) and reduces plasmid size; with no induction step required, it also reduces the number of subcultures performed and the risk of SNP introduction. Overall, the method is fast and results in high editing efficiencies.

## Data Availability

The raw data supporting the conclusion of this article will be made available by the authors, without undue reservation.
