# Supplementary material for: Endogenous CRISPR/Cas systems for genome engineering in the acetogens Acetobacterium woodii and Clostridium autoethanogenum
Source: Front Bioeng Biotechnol. 2023 Jun 23;11:1213236. doi: 10.3389/fbioe.2023.1213236 (PMC10328091; doi:10.3389/fbioe.2023.1213236)
Supplement: Supplementary file 1 [file DataSheet1.pdf]

![frontiers]

Supplementary Material

Endogenous CRISPR/Cas systems for genome engineering in the acetogens *Acetobacterium woodii* and *Clostridium autoethanogenum*.

Margaux Poulalier Delavelle*, Jonathan P. Baker, James Millard, Klaus Winzer and Nigel P. Minton*

***Correspondence:** Corresponding Authors: Nigel P. Minton (nigel.minton@nottingham.ac.uk) and Margaux Poulalier Delavelle (mbxmp6@exmail.nottingham.ac.uk)

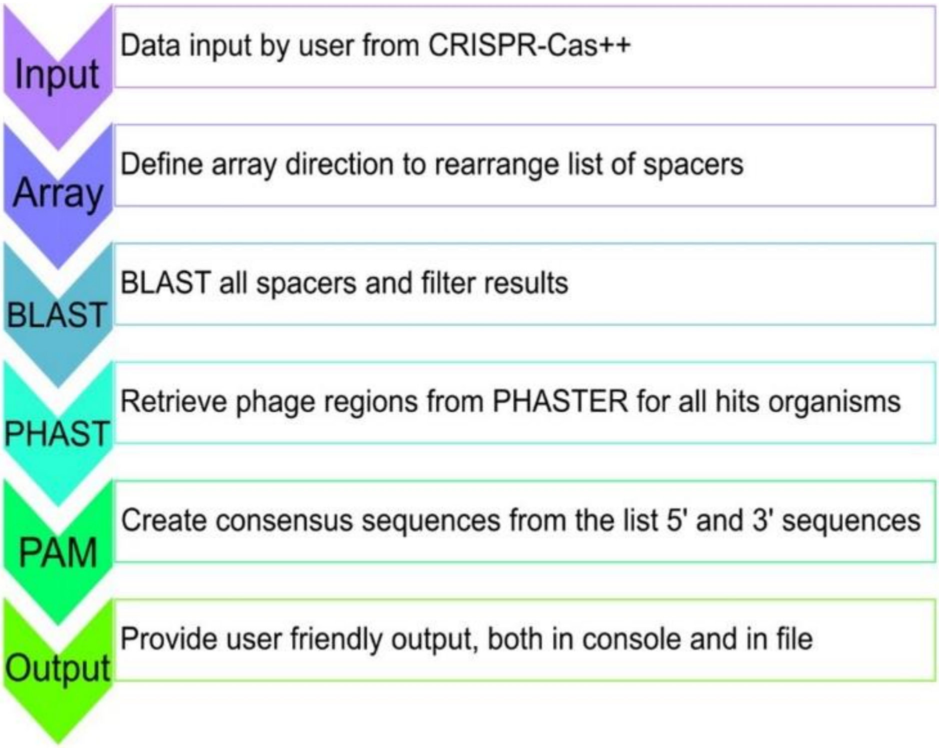

Supplementary Figure S1: General overview from initial user input to final user-friendly results of the developed Python script.

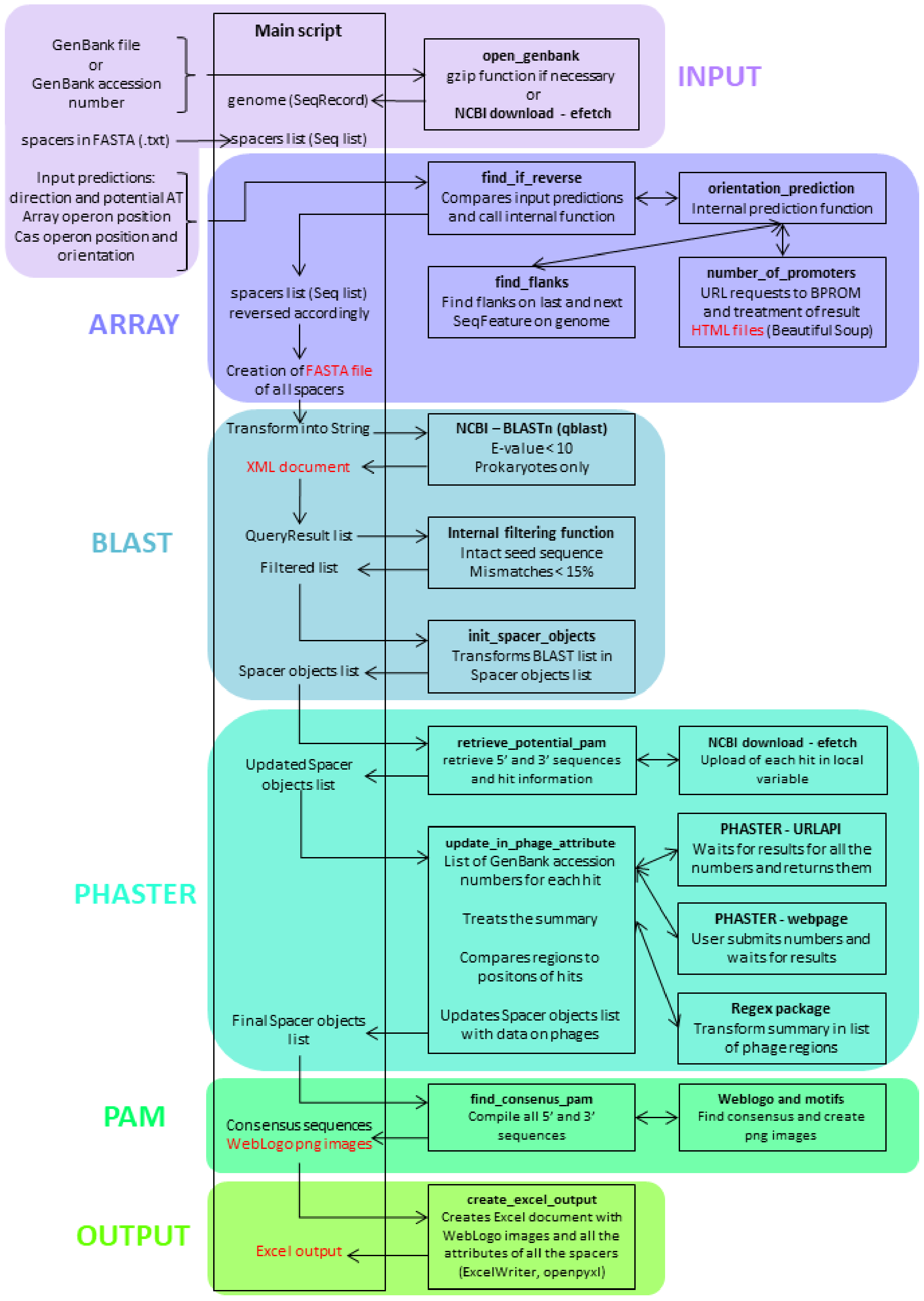

Supplementary Figure S2: Schematic representation of all the steps of the Python script as described in this study. The colour code for each block references Figure 6. The outputs saved to the working directory are written in red, they allow the user to restart the script without submitting again to the different databases or tools, the functions and objects taken from external packages are indicated.

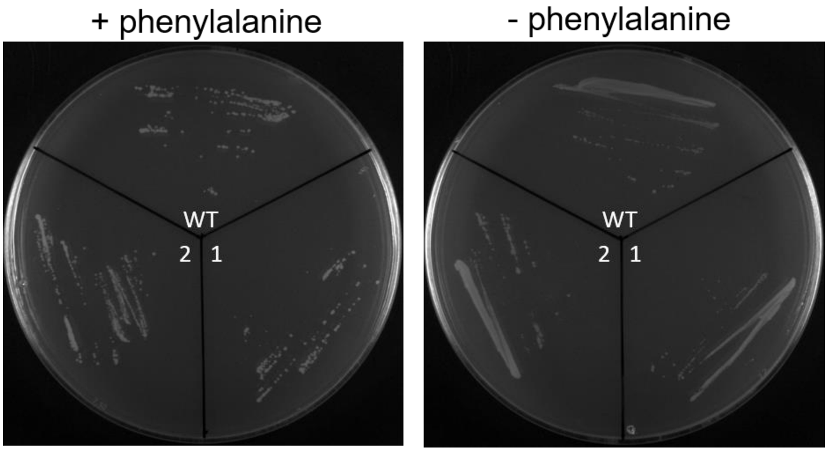

Supplementary Figure S3: Growth on minimal media with and without phenylalanine supplementation (20 µg/mL) of two *A. woodii pheA* KI colonies (marked 1 and 2) in comparison to WT.

Supplementary Table S1: Attributes kept in the Spacer object for each hit during the Python script run.

**Column name**	**Details**
number	Spacer identification number
query	Query number from the original spacer list
iterations	Number of duplicate matches with the same attributes
sequence	Full sequence of the query
target_acc	GenBank accession number of the hit organism found with BLAST
target_org	NCBI name of the hit organism
query_pos	Position of the hit in the query sequence
hit_pos	position of the query in the hit sequence
hit_direc	Direction of the hit, 1 for forward and -1 for reverse
hit_sequence	Sequence of the hit
similarity	Similarity between hit and query sequences, each bar represents a nucleotide match
mismatch	Mismatch percentage between query and hit
hit_locus	Locus in the target genome where the hit was found
hit_product	Product from the gene where the hit was found
in_phage	Indicates if the match was found to be in a phage sequence by PHAST
phage_completeness	Completeness of the phage as indicated by PHAST
phage_name	Phage name as indicated by PHAST
fivep	5′ sequence retrieved from the target organism
threep	3′ sequence retrieved from the target organism

Supplementary Table S2: Bacterial strains

**Bacterial strain**	**Genotype**	**Source**
Escherichia coli TOP10	*mcrA, Δ(mrr-hsdRMS-mcrBC), Phi80lacZ(del)M15, ΔlacX74, deoR, recA1, araD139, Δ(ara-leu)7697, galU, galK, rpsL(Sm^R), endA1, nupG*	Invitrogen
Acetobacterium woodii DSM1030	Wild type strain	DSMZ
Clostridium autoethanogenum DMS10061	Wild type strain	DSMZ
Acetobacterium woodii Δ*pheA* 1.0 kb 5 h	Δ*pheA*, obtained with 1.0 kb HAs and 5 h recovery	This study
Acetobacterium woodii Δ*pheA* 1.0 kb 7.5 h	Δ*pheA*, obtained with 1.0 kb HAs and 7.5 h recovery	This study
Acetobacterium woodii Δ*pheA* 1.5 kb 5 h	Δ*pheA*, obtained with 1.5 kb HAs and 5 h recovery	This study
Acetobacterium woodii Δ*pheA* 1.5 kb 7.5 h	Δ*pheA*, obtained with 1.5 kb HAs and 7.5 h recovery	This study
Acetobacterium woodii Δ*hsdR1* colonies 1 to 3	Δ*hsdR1*	This study
Acetobacterium woodii P_{thl}_FAST	P_{thl}_FAST inserted at *pheA* locus	This study
Clostridium autoethanogenum Δ*pyrE* colonies 1 to 6	Δ*pyrE*	This study

Supplementary Table S3: Rich *A. woodii* media: liquid AWM and modified ATCC medium 1019

***Acetobacterium woodii* liquid medium (AWM), for 1 L**		**Modified ATCC 1019 plates, for 1 L**	
K_2HPO_4	0.45 g	K_2HPO_4	0.4 g
KH_2PO_4	0.33 g	KH_2PO_4	0.4 g
NH_4Cl	1 g	NH_4Cl	1.0 g
NaCl	20 mg	NaCl	0.1 g
$MgSO_4.7H_2O$	0.16 g	$MgSO_4.7H_2O$	0.1 g
$CaCl_2$	20 mg		
yeast extract	2 g	yeast extract	1.0 g
SL9	1 ml	SL9	1 ml
selenite-tungstate	1 ml	selenite-tungstate	1 ml
vitamin solution	20 ml	vitamin solution	2 ml
$NaHCO_3$	10.0 g	$NaHCO_3$	3.0 g
L-cysteine*	0.04% (w/v)	L-cysteine*	40 µl / plate
Fructose*	20 mM	Fructose*	20 mM
Adjust pH to 7.2 with HCl after autoclaving and filter sterilise before adding fructose and L-cysteine. Asterisks indicate elements added after autoclaving.		Add 15 g/L of agar to make plates. Asterisks indicate elements added after autoclaving. Fructose is added when pouring the plates and L-cysteine is spread on the plates before use.	

**Trace element solution SL9, for 1 L**	
nitrilotriacetic acid	12.8 g
$FeCl_2.4H_2O$	2.0 g
$ZnCl_2$	0.070 g
$MnCl_2.4H_2O$	0.1 g
H_3BO_3	0.006 g
$CoCl_2.6H_2O$	0.19 g
$CuCl_2.2H_2O$	0.002 g
$NiCl_2.6H_2O$	0.024 g
$Na_2MoO_4.2H_2O$	0.036 g
First dissolve nitrilotriacetic acid and adjust pH to 6.0 with NaOH, then add minerals. Filter sterilise and store at 4°C.	

**Vitamin solution, for 1 L**	
Biotin	2.00 mg
Folic acid	2.00 mg
Pyridoxine-HCl	10.00 mg
Thiamine-HCl.2H_2O	5.00 mg
Riboflavin	5.00 mg
Nicotinic acid	5.00 mg
D-Ca-pantothenate	5.00 mg
Vitamin B12	0.10 mg
p-Aminobenzoic acid	5.00 mg
Lipoic acid	5.00 mg
Filter sterilise and store at 4°C.	

**Selenite-tungstate solution, for 1 L**	
NaOH	0.5 g
$Na_2SeO_3.5H_2O$	3 mg
$Na_2WO_4.2H_2O$	4 mg
Filter sterilise and store at 4°C.	

Supplementary Table S4: Rich *C. autoethanogenum* YTF media

**YTF, for 1 L**	
Yeast extract	10 g
Tryptone	16 g
Fructose	10 g
NaCl	0.2 g
Acidic trace elements solution	1 ml
Basic trace elements solution	1 ml
Vitamin solution	1 ml
Adjust pH to 5.8 with HCl and autoclave. For agar media, add 15 g of bacteriological agar.	

**Vitamin solution, for 1 L**	
p-aminobenzoate	100 mg
riboflavin	100 mg
thiamine	200 mg
nicotinate	200 mg
pyridoxin	500 mg
pantothenate (calcium)	100 mg
cyanocobalamin	100 mg
d-biotin	20 mg
folate	50 mg
lipoate/thioctic acid	50 mg
2-mercapto sulfonic acid	-
Dissolve p-aminobenzoate separately by titrating with 5 M NaOH until dissolved. Dissolve riboflavin separately at 50°C (0.012% solubility). Dissolve lipoate separately by titrating with 5 M NaOH until dissolved.<br><br>Final pH 7.8; filter sterilise and store at 4°C	

**Acidic trace elements solution, for 1 L**	
HCl	50 mM
H_3BO_3	100 mg
$MnCl_2.4H_2O$	230 mg
$FeCl_2.4H_2O$	780 mg
$CoCl_2.6H_2O$	103 mg
$NiCl_2.6H_2O$	602 mg
$ZnCl_2$	78 mg
$CuSO_4.5H_2O$	50 mg
$AlK(SO_4)_2.12H_2O$	50 mg
Filter sterilise and store at 4°C.	

**Basic trace elements solution, for 1 L**	
NaOH	10 mM
Na_2SeO_3	58 mg
Na_2WO_4	53 mg
$Na_2MbO_4.2H_2O$	52 mg
Filter sterilise and store at 4°C.	

Supplementary Table S5: Minimal *C. autoethanogenum* PETC MES media

**PETC MES minimal, for 1 L**	
NH_4Cl	1.00 g
KCl	0.10 g
$MgSO_4.7H_2O$	0.20 g
KH_2PO_4	0.20 g
$CaCl_2$	0.02 g
Nitrilotriacetic Acid	0.05 g
$Fe(SO_4)_2(NH_4)_2$.6H2O	0.05 g
CH_3COONa	0.25 g
MES buffer	20.00 g
Trace metals (100X)	10 ml
Wolfe's vitamins (100X)	10 ml
Resazurin (2 $g.L^{-1}$) (2000X)	0.5 ml
Adjust pH to 5.8 with NaOH and autoclave. For agar media, add 15 g of bacteriological agar.	

**Wolfe's vitamin solution, for 1 L**		**100X Trace metal solution, for 1 L**	
Biotin	0.004 g	Nitrilotriacetic Acid	2.00 g
Folic acid	0.004 g	$MnSO_4.H_2O$	1.00 g
Pyridoxine hydrochloride	0.002 g	Fe $(SO_4)_2(NH_4)_2.6H_2O$	0.80 g
Thiamine.HCl	0.010 g	$CoCl_2.6H_2O$	0.20 g
Riboflavin	0.010 g	$ZnSO_4.7H_2O$	0.0002 g
Nicotinic acid	0.010 g	$CuCl_2.2H_2O$	0.02 g
Calcium D-(+)-pantothenate	0.010 g	$NaMoO_4.2H_2O$	0.02 g
Vitamin B_{12}	0.0002 g	Na_2SeO_3	0.02 g
p-Aminobenzoic acid	0.010 g	$NiCl_2.6H_2O$	0.02 g
Thioctic acid	0.010 g	$Na_2WO_4.2H_2O$	0.02 g
Filter sterilise and store at 4°C.			

Supplementary Table S6: Primers

	Primer name	Sequence	Description
pMTL80000 series	ColE1+tra-F2	CCATCAAGAAGAGCGAC	Used for colony PCRs and Sanger sequencing of vectors
	pBP1-R1	CTTCATTAAATGCCTTAGAATC	
	pCB102-R1	CTGTTATGCCTTTTGACTATC	
	pCD6-R1	GACTTTAAGCCTACGAATACC	
A. woodii interference assay	FW_proto8	AAACAGCTATGACCgcggccctagaCAAATGAATCAAAATCATTAATAGCTTTAATTAAAT	Assembly of each of the 3 protospacer with and without 5′ PAM
	RV_proto8	gcgcgccattGACGTCtatGTCGACTAAATGACGATTTAATTAAAGCTATTAATGATTTTGA	
	FW_proto8-5′	AAACAGCTATGACCgcggccctagaATGCCGAATCAAAATCATTAATAGCTTTAATTAAAT	
	FW_proto20	AAACAGCTATGACCgcggccctagaCAAATTTATTCTATGAGGTGATGAGATGAATGTT	
	RV_proto20	cgccattGACGTCtatGTCGACTAAATTCTTAATAACATTCATCTCATCACCTCAT	
	FW_proto20-5′	AAACAGCTATGACCgcggccctagaGGGTGTTATTCTATGAGGTGATGAGATGAATGTT	
	FW_proto22	AAACAGCTATGACCgcggccctagaCAAATAATAACTATCAGGCTATCAGCTTCGGGG	
	RV_proto22	ccattGACGTCtatGTCGACTAAATAAAGTCTCCCCGAAGCTGATAGCCTGATA	
	FW_proto22-5′	AAACAGCTATGACCgcggccctagaAACCAAATAACTATCAGGCTATCAGCTTCGGGG	
Leader characterisation	FW_leader	CAGGAAACAGCTATGACCgcggttgtcgacctgtgat	Assembly of leader_MCS
	RV_leader_SacI	atatggatctactcaaatgagctcccgtgcttgttagatacc	
	FW_catP_SacI	gcacgggagctcatggtatttgaaaaaattg	Assembly of leader sequence with catP or FAST gene
	RV_catP_NheI	gcaggaattgataaatagttaagctagcataaaa	
	FW_FAST_SacI	gcataagagctcATGGAACACGTAGC	

	RV_FAST_NheI	atttttatgctagcTCATACCCTCTTAACG	
A. woodii pyrE KO	FW_LHA_pyrE	ggcttcttatttttatGACGTCTAGCAACTGGGAAGATGCG	Amplification of HAs from gDNA
	RV_LHA_pyrE+BM4	CCTTCCGTACAACCCACAACCCTTTTTCGTTCCGTAAAAATGG	
	FW_BM4+1+RHA_pyrE	AGGGTTGTGGGTTGTACGGAAGGATAAGTTAAAGAGCACGTAAA	
	RV_RHA_pyrE	aataatggcggcgcgccGCTGGTAACATGAATGC	
	FW_leader	CAGGAAACAGCTATGACCGCGGTTGTCGACCTGTGAT	Amplification of leader sequence
	RV_leader	ATCTACTCAAATGTCGACCCGTGCTTGTTAGATACCTAT	
	RV_RHA_pyrE+MCS	tcttatttttatgctagcGCTGGTAACATGAATGC	RV editing cassette
	FW_pyrE_endo2	tctaacaagcacggGTCGACatttgagtagatccatattggaatgtaaataggcgatcttacccgcttgacaaaccgggcaatcgt	spacer assembly with flanking DR
	RV_pyrE_endo2	CGCATCTTCCCAGTTGCTAGACGTCatttacattccaatatggatctactcaaatacgattgcccggtttgtcaagcgggtaagat	
	AW_CRISPR_pyrE_armF	tatacgcgcgcggatatcccggttatggcgat	Colony PCR for *pyrE* locus
	AW_pyrEcomp_RHAR	tataGGCGCGCCccatccattttcatctgatt	
A. woodii pheA KO	FW_pheA_spacer	taacaagcacgggagctcatttgagtagatccatattggaatgtaaataaccaatgtcacccgatttgtgattatttcccgca	spacer assembly with flanking DR
	RV_pheA_spacer	ccatggacgcgtgacGTCatttacattccaatatggatctactcaaattgcgggaaataatcacaaatcg	
	RV_LHA_pheA	taatctggaaATTCTTtgacttaaaatcttctc	LHA and RHA amplification, lengths of 0.5 kb, 1.0 kb and 1.5 kb
	FW_RHA_pheA	taagtcaAAGAATttccagattatagatttccccggc	
	FW_L0.5	aaatGACgtccggcacagtcgatgttag	
	RV_R0.5	tatgctagctctatccgcaaacgctt	
	FW_L1.0	aaatGACgtcattgacggctgggg	

	RV_R1.0	atgctagcgagattggcctgctc	
	FW_L1.5	taaatGACgtccagccgactgatactga	
	RV_R1.5	ttttatgctagcaagcctcgtcatcgc	
	FW_pheAHA1.5_out	ccggaaagggatcaaatttg	Colony PCR primers
	RV_pheA HA1.5_out	tggtttcgtctttttaggtag	
A. woodii hsdR1 KO	FW_hsdR1_spacer	aacaagcacgggagctcatttgagtagatccatattggaatgtaaattcgaaatactgaccggcctgggctac	spacer assembly with flanking DR
	RV_hsdR1_spacer	ctgtatttgaatttcattGACGTCatttacattccaatatggatctactcaaataggcatacgcgtagcccaggccggtcagtatt	
	FW_LHA_hsdR1	gtaaatGACGTCGGTTGTGATAGAGCTT	LHA and RHA amplification
	RV_LHA_hsdR1	ctgctacacccgtaaactcataacgtgtccc	
	FW_RHA_hsdR1	gagtttacgggtgtagcaggttg	
	RV_RHA_hsdR1	tatttttatgctagcaagtgttcacc	
	FW_hsdR1_screen_1.5	GCTGTTGATGATGGGATG	Colony PCR primers
	RV_hsdR1_screen_1.5	cattggcatagggatttttc	
	FW_hsdR1_del_screen	caataaagtaacgggttgatta	Deletion sequencing
	RV_hsdR1_del_screen	caagtatgctatgattgtatc	
A. woodii pheA CCG KI	FW_AWO_pheA_CCG_KI_spacer	aagcacgggagctcatttgagtagatccatattggaatgtaaatcttttttgaccttaagcagtcgtcgttaaagcggggatttgagta	spacer assembly with flanking DR
	RV_AWO_pheA_CCG_KI_spacer	gcaaaaataaccctcgaGACGTCatttacattccaatatggatctactcaaatccccgctttaacgacgactgcttaaggtcaa	
	FW_AWO_LHA_pheA_cargo	gtaaatGACGTCtcgagggttatttttgcaa	LHA and RHA amplification
	RV_AWO_LHA_pheA_CCG_cargo_MCS	gaatcgtcatatggacagcggccgcgactcGGATCCggggttatctgtcatg	

	FW_AWO_RHA_pheA_CCG_cargo_MCS	gccgctgtccatatgacgattctagacggctttttgaccttaagcagtcgtc	
	RV_AWO_RHA_pheA_cargo	tttatgctagccagccgactgatactgaa	
	FW_BamHI_FAST_cassette	gataaccccGGATCCtttttaacaaaatatattgataaaaat	Cargo amplification P_{thl}_FAST_term
	RV_FAST_NotI_AscI	ttaagggcgggcgcgccaGCGGCCGCTCATACCCTCTTAACGAA	
	FW_pheA_KI_seq	gtcaatgattcatcgagttc	Colony PCR and Sanger sequencing primers
	RV_pheA_KI_seq	gataattatcagaatcttagtaaatc	
	FW_pheA_cargo_seq	CTTCACTGGTAATATTTCAC	
	RV_pheA_cargo_seq	TGATTATAGCCCGGTAGTAGA	Vector Sanger sequencing
C. autoethanogenum pyrE KO	FW_leader4_CLAU	ctatgaccgcggaatacacattctccctcat	Amplification of the leader sequence of CRISPR array4
	RV_leader4_CLAU	GGTACCGAGCTCCACAGTAAAATAGCCATTTCT	
	FW_CLAUpyrE_HA	ccggggatccgtaccgctg	Amplification of the assembled HAs from vFS67
	RV_CLAUpyrE_HA	tttatgctagccctgtaatcggagcatc	
	FW_CLAUpyrE_spacer1	atggctattttactgtggagctcGTTGAACCTCAACATGAGATGTATTTAAATctgtgttttcctgaagaaagtagaaaatg	spacer assembly with flanking DR
	RV_CLAUpyrE_spacer1	cgcgtgacgtcgactctagaggatccATTTAAATACATCTCATGTTGAGGTTCAACgaagggcattttctactttcttcaggaaaa	
	FW_pyrECLAU_screening	gttaattgagaatcttttgctattac	Colony PCR primers
	RV_pyrECLAU_screening	gtcttgtatgtacttgtagtac	

Supplementary Table S7: Plasmids

	Plasmid name	Description	Source
Backbones	pMTL82151	Cm^R; pBP1	SBRC Nottingham culture collection
	pMTL84151	Cm^R; pCD6	
	pMTL8415X_P*thl*_FAST	Cm^R; pCD6; P*thl*_FAST	
	pRECas1_MCS	Cm^R; P*fdx*-RbE-Cas9; P_{araE}-MCS; pCB102	Canadas et al. 2019
	pMTL8215_P1339_MCS	Cm^R; P*araE*-MCS; pBP1	This study
	pMTL-MPD15	Cm^R; leader sequence upstream a MCS; pCD6	This study
	pMTL-MPD23	Cm^R; leader sequence upstream *pheA* intergenic spacer; *pheA* HAs flanking a MCS; pCD6	This study
	pMTL-MPD25	Cm^R; *C. autoethanogenum* leader4 sequence upstream a MCS; pCB102	This study
A. woodii interference assay	pMTL-MPD1	Cm^R; pBP1; *A. woodii* protospacer 8	This study
	pMTL-MPD2	Cm^R; pBP1; *A. woodii* protospacer 8 with 5′ PAM candidate	This study
	pMTL-MPD3	Cm^R; pBP1; *A. woodii* protospacer 20	This study
	pMTL-MPD4	Cm^R; pBP1; *A. woodii* protospacer 20 with 5′ PAM candidate	This study
	pMTL-MPD5	Cm^R; pBP1; *A. woodii* protospacer 22	This study
	pMTL-MPD6	Cm^R; pBP1; *A. woodii* protospacer 22 with 5′ PAM candidate	This study
Leader characterisation	pMTL-MPD29	Cm^R; pCD6; leader_catP	This study
	pMTL-MPD30	Cm^R; pCD6; leader_FAST	This study
A. woodii pyrE KO	pMTL-MPD7	Cm^R; P*araE*-MCS; *pyrE* HAs; pBP1	This study
	pMTL-MPD8	Cm^R; leader sequence upstream *pyrE* spacer2; *pyrE* HAs; pBP1	This study

	pMTL-MPD9	Cm^R; *pyrE* HAs; pBP1	This study
	pMTL-MPD10	Cm^R; leader sequence upstream *pyrE* spacer2; pBP1	This study
	pMTL-MPD11	Cm^R; leader sequence upstream *pyrE* spacer2; *pyrE* HAs; pBP1	This study
	pMTL-MPD12	Cm^R; *pyrE* HAs; pCD6	This study
	pMTL-MPD13	Cm^R; leader sequence upstream *pyrE* spacer2; pCD6	This study
	pMTL-MPD14	Cm^R; leader sequence upstream *pyrE* spacer2; *pyrE* HAs; pCD6	This study
A. woodii pheA KO	pMTL-MPD16	Cm^R; *pheA* 0.5 kb HAs; pCD6	This study
	pMTL-MPD17	Cm^R; *pheA* 1.0 kb HAs; pCD6	This study
	pMTL-MPD18	Cm^R; *pheA* 1.5 kb HAs; pCD6	This study
	pMTL-MPD19	Cm^R; leader sequence upstream *pheA* spacer; *pheA* 0.5 kb HAs; pCD6	This study
	pMTL-MPD20	Cm^R; leader sequence upstream *pheA* spacer; *pheA* 1.0 kb HAs; pCD6	This study
	pMTL-MPD21	Cm^R; leader sequence upstream *pheA* spacer; *pheA* 1.5 kb HAs; pCD6	This study
A. woodii vectors	pMTL-MPD22	Cm^R; leader sequence upstream *hsdR1* spacer; *hsdR1* HAs; pCD6	This study
	pMTL-MPD24	Cm^R; leader sequence upstream *pheA* intergenic spacer; *pheA* HAs flanking P_{thl}_FAST; pCD6	This study
C. autoethanogenum pyrE KO	pMTL-MPD26	Cm^R; *pyrE* HAs; pCB102	This study
	pMTL-MPD27	Cm^R; *C. autoethanogenum* leader4 sequence upstream *pyrE* spacer; pCB102	This study
	pMTL-MPD28	Cm^R; *C. autoethanogenum* leader4 sequence upstream *pyrE* spacer; *pyrE* HAs; pCB102	This study

Supplementary Table S8: Primers for RNA quality control and RT-qPCR

Primer name	Sequence	Product size	Comments
gDNA_FW	tatacgcgcgcggatatcccggttatggcgat	1997 bp	gDNA contamination control
gDNA_RV	tataGGCGCGCCccatccattttcatctgatt		
FWD 4	GGAAGGAGTAGCTAGTGGTAAT	145 bp	FAST reporter gene
REV4	CCTCTTAACGAAAACCCAGTAG		
Chow_gyrA_FW	GTAAGTCGGCCCGTATTGTTG	108 bp	*A. woodii* gyrase, internal reference gene (Shin et al., 2021, Chowdhury et al., 2020)
Chow_gyrA_RV	AACGGATCGACCATTCCTGAG		
orfB_FWD	gtattacacaggagtttgaacc	74 bp	Plasmid backbone.
orfB_REV	aaatactctgcttggtcggctt		

Supplementary Table S9: Comparison of the 5′ PAM consensus sequences (5′ → 3′) found in the literature with the ones from the Python script for different strains of *Clostridium spp.*. Bold: PAMs verified experimentally; *: sequence that have not been experimentally tested. The total indicates the number of spacers analysed; Hits in phage, the number of spacers that were found in invading elements, all the consensus were done by WebLogo 3.7.4 (Crooks et al., 2004). (Ambiguous nucleotides codes: W for A or T; R for A or G; K for T or G; D for A, G or T)

**Strain**	**Total**	**Hits in phage**	**Script WebLogo**	**Script consensus 5′ → 3′**	**Consensus (source) 5′ → 3′**
C. difficile 630	105	234	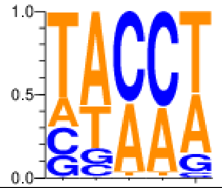	CCW / AAK*	**CCW** (Boudry et al., 2015, Maikova et al., 2019)
C. difficile R20291	96	147	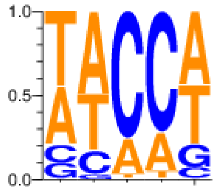	CCW	**CCW** (Boudry et al., 2015, Maikova et al., 2019)
C. pasteurianum ATCC 6013	45	18	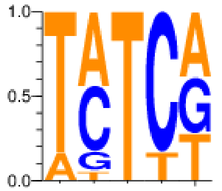	TCW / TTG / TCG* / TTA*	**TCW / TTG** (Pyne et al., 2016)
C. tetani 12124569	31	14	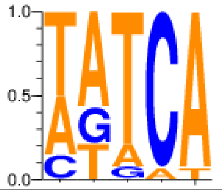	TCA	TNA (Pyne et al., 2016)
C. thermocellum ATCC 27405	168	5	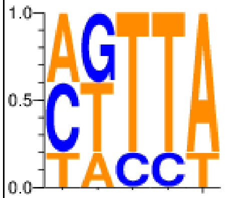	TTA / TTG / TCA	NCA (Pyne et al., 2016) **TTN / TNA** (Walker et al., 2020)
C. tyrobutyricum	25	4	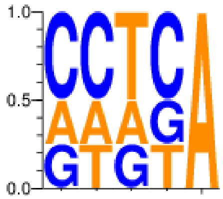	TCA / GGA* / ATA*	**TCR** (Zhang et al., 2018)

Supplementary Table S10: Details of the analyses of the arrays corresponding to CRISPR/Cas Type I-B systems of a range of *Clostridium spp.*. The array numbers correspond to the ones that can be found in the CRISPRFinder database. The number of spacers found in invading elements corresponds to the number found by the script to be in plasmid / phages / prophages / mobility-related genes. The 5′ PAMs indicated were compiled manually to account for separate consensus sequences possible for each organism. . (Ambiguous nucleotides codes: Y: C or T; S: G or C; M: A or C; W: A or T; D: not C; H: not G)

Strain	Array	# of spacers	# in invading elements	5′ WebLogo	5′ PAM 5′→ 3′
C. butyricum KNU-L09	3	57	14	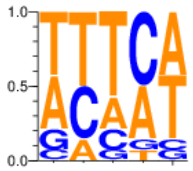	TCM / TAW / GTT / CCG / TGT / CAT / ACT / AAA
C. limosum 14S0207	1	29	8	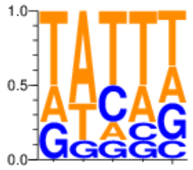	unclear
	2	35	26	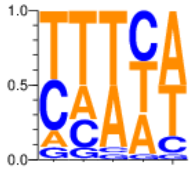	TCT / ATA / TAA / ATT
	3	40	3	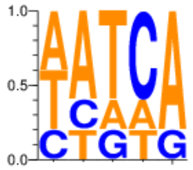	GTG / TCA / AAA
C. novyi NT	2	20	8	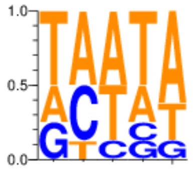	TTD / GYA / TCA / SAC / AAG / CCA
	3	26	10	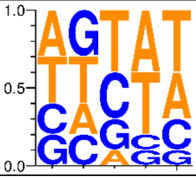	HAT / TTM / GTW / CGC / TCA
	4	50	12	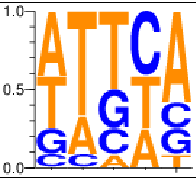	TTT / AAA / TGG / CCA / ATA

Supplementary Table S11: List of protospacer matches in invading elements from *A. woodii* spacers obtained with the Python script described. # is the position of the spacer in the array; the spacer and protospacer sequences are aligned and mismatches underlined; % is the percentage of mismatch between spacer and protospacer sequences; the invading elements are indicated and position of the hit in the genome indicated if relevant in parentheses; potential 5′ PAM are indicated in the last column based on each match.

#	Spacer – protospacer sequences	%	Invading element	5′ PAM
5	GGGAGGAATAAAAATGAACTTAAAAGATGTAAAAAG<br>-GGAGGAATCAAAATGAACTTAAAAAATTTAAA---	19	*Fusobacterium varium* Fv113-g1 (questionable prophage)	AAATA
5	GGGAGGAATAAAAATGAACTTAAAAGATGTAAAAAG<br>-GGAGGACTAAAAATGAACTTAAATGATGAACAAA-	17	Bacteriophage sp. isolate 150	CTATA
5	GGGAGGAATAAAAATGAACTTAAAAGATGTAAAAAG<br>-GGAGGATTAAAAATGAACTGTAAAGAATTTAAAAG	19	*Fusobacterium* sp. oral taxon 203 (incomplete prophage)	AATAA
8	GAATCAAAATCATTAATAGCTTTAATTAAATCGTC<br>GAATCTAAATTTTTAATAGCTTTTATCACATCGTC	17	*Yersinia* phage phiR1-RT	ATGCC
15	TTGAAGACATGAGAAAAG---CAAAAGAAACAATTCTT<br>TTGAAGAAATGAGTAACGTTCCAAAAGAAACAATT---	17	*Listeria monocytogenes* FDAARGOS_57 (unnamed plasmid)	AACTT
29	TTATTCTATGA--GGTGATGAGATGAATGTTATTAAGA<br>TTATTCTATCATTGGTGATAAGATGAATGTTACTA---	17	*Aeromonas* phage AS-zj	GGGTG
34	AATAACTATCAGGCTATCAGCTTCGGGGAGACTTT<br>AATAACTATTATGCTATCAGCTTCAGGCATACT--	20	*Hathewaya histolytica* NCTC503, (phage protein)	AACCA
45	ACCACCAACGCCGGGATTACCCTTGCAAGCGCCATC<br>-CCACCAGGGCCTGGATTACCCTTGCACGCACCATC	17	*Pseudomonas* sp. Leaf58 pBASL58	CCCAG

Supplementary Table S12: List of protospacer matches in invading elements from *C. autoethanogenum* spacers obtained with the Python script described in increasing mismatch percentage (up to 15%). # refers to the array number and the position of the spacer in the array; the spacer and protospacer sequences are aligned and mismatches underlined; % is the percentage of mismatch between spacer and protospacer sequences; the invading elements are indicated and position of the hit in the genome indicated if relevant in parentheses; potential 5′ PAM are indicated in the last column based on each match.

#	Spacer – protospacer sequences	%	Invading element	5′ PAM
4-27	GCACCTAAGTAATCTATAAGTAAAGTATCAACTCTT<br>GCACCTAAGTAATCTATAAGTAAAGTATCAACTCTT	0	*Clostridium ljungdahlii* DSM 13528 (phage protein)	CTTCA
4-27	GCACCTAAGTAATCTATAAGTAAAGTATCAACTCTT<br>GCACCTAAATAATCTATAAATAAAGTTTCAACTCTT	8	incomplete phage PHAGE_Staphy_PT1028_NC_007045	CATCA
4-8	CCTCATATTCTTTTAATAATTTATAAAATGTATTT<br>-CTCATATTCTTTTATTATTTTATAAAATGTATTT	9	*Bacillus cereus* strain FORC087 plasmid pFORC087.2	GTGTT
4-7	TTTCTCTTAT-AGATCCTTTAATTCTTCTATAGCTTC<br>TTTCTCTTATTAAACCCTTTAATTCTTCTAAATCTTC	11	intact phage PHAGE_Clostr_phiCT19406C_NC_029006	TTCCC
4-8	CCTCATATTCTTTTAATAATTTATAAAATGTATTT<br>CCTCATATTCTTTTATTATTTTATAGAAAGTATTT	11	*Bacillus thuringiensis* strain YGd22-03 plasmid pYGD30 (recombinase gene)	TTTTA

4-17	TCTTTTGATATGGCCCCTGCATGAGAAAGTTGTTG<br>TCTTTTGAAATGACACCTGCATGAGAAAGCTGTTG	11	intact phage PHAGE_Clostr_phiCT9441A_NC_029022	TTGCA
4-10	AATACATCTATATGCTTAATGCATGTAGTATCTATAC<br>AATATATCTATATGCTTAATACATGTAGTATCTA---	14	*Clostridium pasteurianum* BC1 (phage portal protein)	TAGAG
3-21	TTTATACATTCCTCCATTTTTATACTAATTATTTAA<br>TTTTTACATTCCTCCAATTTTTTATTTATTATTTAA	14	intact phage PHAGE_Clostr_phiCTC2B_NC_030951	ACTCA
3-22	TGGAAAGGAAGTATA-ATATGATAGATGAGATATTAA<br>-GGAAAGGATGTATAAATATGATAGATAAAATAATAA	14	intact phage PHAGE_Clostr_phiCD27_NC_011398	ATAAA
3-25	CTTACAGGCGTATGTCCAAGATCATTTACTGCCTCC<br>CTTACAGGAGCATGTCCAAGGTCATTCACTGCTTCC	14	incomplete phage PHAGE_Clostr_phiCTC2A_NC_030949	CTTCA
4-27	GCACCTAAGTAATCTATAAGTAAAGTATCAACTCTT<br>GCACCTAAATAATCTATAAATAATGTGTCAACTCT-	14	questionable phage PHAGE_Clostr_vB_CpeS_CP51_NC_021325 (virulence-associated protein E)	CTTCA
4-28	GAATTTA-TATAAACCCAATCAGAATATATATAATTA<br>GAATATACTATAAAACCAATTACAATAGATATAATTA	14	*Clostridium taeniosporum* strain 1/k plasmid pCt3	CTGAT

4-28	GAATTTA-TATAAACCCAATCAGAATATATATAATTA<br>GAATATACTATAAAACCAATTACAATAGATATAATTA	14	*Clostridium taeniosporum* strain 1/k plasmid pCt3	CTGAT
4-6	TATATATCTATACCTTTTAACAATTTAAATACCTC<br>-ATATATCTATTCCTTTTAAC-ATTTACATTCCTC	14	questionable phage PHAGE_Clostr_phiMMP04_NC_019422	ATTTG
4-8	CCTCATATTCTTTTAATAATTTATAAAATGTATTT<br>CCTTATATTCTTTCAATAATTTATCATATGTTTTT	14	*Borrelia turicatae* strain BTE5EL plasmid lp159	ATATT

Supplementary Tables S13: List of protospacer matches in invading elements from *C. autoethanogenum* spacers obtained with the Python script described in increasing mismatch percentage (between 15% and 20%). # refers to the array number and the position of the spacer in the array; the spacer and protospacer sequences are aligned and mismatches underlined; % is the percentage of mismatch between spacer and protospacer sequences; the invading elements are indicated and position of the hit in the genome indicated if relevant in parentheses; potential 5′ PAM are indicated in the last column based on each match.

#	Spacer – protospacer sequences	%	Invading element	5′ PAM
3-37	GTGGATTTAGGATCTTTTGTGAAGTTTCTATTTATAA<br>-TGGATT-AGGCTATTTTGT--AGTTTCTATTTATAA	16	incomplete phage PHAGE_Staphy_PT1028_NC_007045	CAAAT
4-10	AATACATCTATATGCTTAATGCATGTAGTATCTATAC<br>AATATATCTAAATGTTTTATACATGTAGTATCTATA-	16	*Clostridium botulinum* CDC_53174, (phage portal protein)	CTGCA
2-9	GAATACAGATACCAATCTCGGAACTGGAATAACTAA<br>-AATACAGATACTAATATTGGAAGTGGAATAAGTAA	17	*Clostridium kluyveri* NBRC 12016 plasmid pCKL1	GATTA
2-20	GAAATTAAAAAGC---TTGAAGAACAAATTCTTGATAGC<br>GAAAGTAAAAAGCCACTTGAAGAACAAATTATTGA----	17	*Mycoplasma salivarium* NCTC10113 plasmid 2	TTCTA
2-20	GAAATTAAAAAGCTT---GAAGAACAAATTCTTGATAGC<br>GAAATTAAAAAGAAATATGAAGAAAAAATTCTTGAT---	17	*Spiroplasma citri* C5 plasmid pScpC5-3	GTACA

3-3	TATATGCCTGTAATTAATAAAATTACTGCTATTATT<br>TATAT-CCT-TAATTAAAAAAATTAGTGCTATTA--	17	incomplete phage PHAGE_Lister_B054_NC_009813	ATTAT
3-7	AAAAAATGACTAATATAATATTATCTTATCCATTAT<br>AAAAAATGACGAATATCATTTTAT-TTATACATTA-	17	*Candidatus Profftella armatura* YCPA plasmid	AATCA
3-10	TGATGTAATTATTCCTAATCCAGTATCTTTAGCACC<br>TGATGTAAAAGTGCCTAATCCAGTAGTTTTAGCACC	17	intact phage PHAGE_Clostr_vB_CpeS_CP51_NC_021325	GTTAA
4-27	GCACCTAAGTAATCTATAAGTAAAGTATCAACTCTT<br>GCACCTAGGTAATCTATTAATAAAGTGTTTACTCTT	17	Clostridium phage phiCT453A (virulence-associated protein E)	CTTCA
2-20	GTTTCTT--TTGTAGGAGTATTCTTTGTAGTGGTTGT<br>-TTTCTTGGTTGTAGTAGTCTTCTTTGTAGTAGTT--	17	uncultured phage genome assembly, NCBI LR745210	GTAGA
4-6	TATATATCTATACCTTTTAACAATTTAAATACCTC<br>-ATATATCTATTCCTTTTATCATTTTACATTCCTC	17	questionable phage PHAGE_Clostr_PhiS63_NC_017978	ATTTG

4-8	CCTCATATTCTTTTAATAATTTATAAAATGTATTT<br>-CTCATATTCTTTCATTACCTTGTAAAATGTATTT	17	*Bacillus pseudomycoides* BTZ plasmid pBTZ_1	TGATT
4-10	AATACATCTATATGCTTAATGCATGTAGTATCTATAC<br>AATAAATCAATGTGCTTAATGGCTTTAGTATCTATA	19	intact phage PHAGE_Lister_2389_NC_003291	CATCT
3-1	GCGAATAGTACCTTTGGTTTTTGCTCCTGGAGCTGC<br>GCAAATATTACTTTTGGCTTTTGCTCCTGAAGTTG-	19	*Clostridium kluyveri* DSM 555 (predicted prophage)	TTTCT
4-7	TTTCTCTTATAGATCCTTTAATTCTTCTATAGCTTC<br>TTTGTCTTAATGATCCTTTAATTCTTTTGTAGCT--	19	intact phage PHAGE_Clostr_vB_CpeS_CP51_NC_021325	TTATT
4-27	GCACCTAAGTAATCTATAAGTAAAGTATCAACTCTT<br>GCGCCTAAGTAATCTATAAGTAAGGTATCAA-----	19	*Clostridium scatologenes* ATCC 25775 (phage-like protein)	CCTCA
4-27	GCACCTAAGTAATCTATAAGTAAAGTATCAACTCTT<br>GCACCTAAATAGTCTATAAATAAAGTACTTACTCT-	19	intact phage PHAGE_Clostr_vB_CpeS_CP51_NC_021325	CTTCC
